# Exploring the Functional Landscape of the p53 Regulatory Domain: The Stabilizing Role of Post-Translational Modifications

**DOI:** 10.1021/acs.jctc.4c00570

**Published:** 2024-07-08

**Authors:** Michael J. Bakker, Oskar Svensson, Henrik V. So̷rensen, Marie Skepö

**Affiliations:** † Faculty of Pharmacy in Hradec Králové, Charles University, Akademika Heyrovského 1203/8, 500 05 Hradec Králové, Czech Republic; ‡ Division of Computational Chemistry, Department of Chemistry, Lund University, P.O. Box 124, 221 00 Lund, Sweden; ¶ NanoLund, Lund University, Box 118, 221 00 Lund, Sweden; § MAX IV Laboratory, Fotongatan 2, 224 84 Lund, Sweden

## Abstract

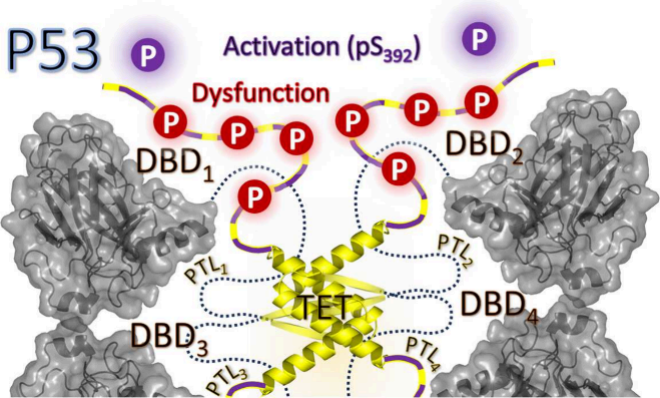

This study focuses on the intrinsically disordered regulatory domain of p53 and the impact of post-translational modifications. Through fully atomistic explicit water molecular dynamics simulations, we show the wealth of information and detailed understanding that can be obtained by varying the number of phosphorylated amino acids and implementing a restriction in the conformational entropy of the N-termini of that intrinsically disordered region. The take-home message for the reader is to achieve a detailed understanding of the impact of phosphorylation with respect to (1) the conformational dynamics and flexibility, (2) structural effects, (3) protein interactivity, and (4) energy landscapes and conformational ensembles. Although our model system is the regulatory domain p53 of the tumor suppressor protein p53, this study contributes to understanding the general effects of intrinsically disordered phosphorylated proteins and the impact of phosphorylated groups, more specifically, how minor changes in the primary sequence can affect the properties mentioned above.

## Introduction

**Figure 1:**
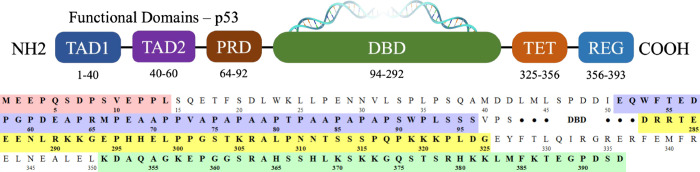
Functionally specific regions, top, of the p53 protein and the amino acid, AA, sequences are described (bottom) with disordered regions highlighted: the trans-activational domain 1 (TAD1)/A-Region in red, the proline-rich domain (PRD)/B-Region in blue, the pretetramerization loop (PTL)/C-Region in yellow, and the C-terminal regulatory domain (REG)/D-Region in green.

The p53 protein is a tumor suppressor protein^1−3^ that plays a crucial role in regulating cell growth^4,5^ and halting the propagation of cancer.^6−8^ Many of p53’s domains are well studied,^9−11^ specific regions are enigmatic due to their intrinsic disorder.^12^ These unstructured regions can be classified into four distinct regions; the trans-activational domain 1, TAD1_(1–14)_, the proline-rich domain PRD_(51–96)_, the pretetramerization loop, PTL_(281–325)_, and the regulatory domain, REG_(350–393)_. The flexibility of each region is of relevance to the specific function it serves. Previously,^13^ we investigated PTL_(281–325)_ for its ability to contribute to tetramerization upon expansion or contraction. Figure 1 displays the specific functional domains and their corresponding amino acid (AA) sequences, with the intrinsically disordered regions (IDRs) colored shaded.

**Figure 2:**
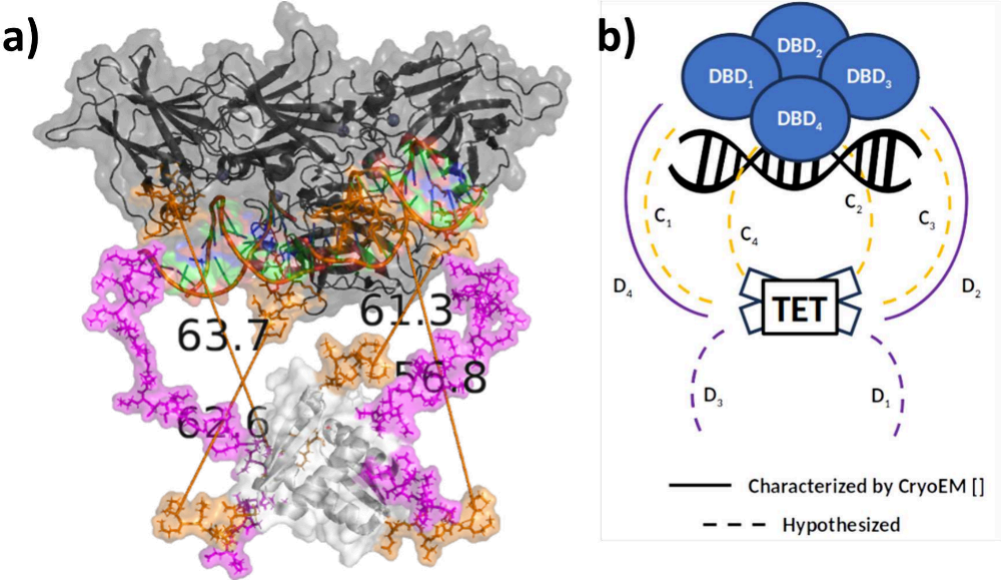
(a) Three-dimensional structure of the p53 tetramer-DNA complex, including the stabilizing tetramerization domain (white) and the missing atomic coordinates in the PTL in orange. The presence of two C-terminal regulatory domain (REG) can also be observed in purple. (b) Schematic representation of the p53 tetramer with identical color coding.

REG_(356–393)_ interacts with other proteins and modulates their function. It permits p53 to interact with a wide range of target proteins,^14^ and controls various cellular processes.^15,16^ It also contains binding sites established for interacting with other molecules, such as phosphatases, enabling post-translational modifications (PTMs).^17−19^ The region contains multiple phosphorylation sites that are of biological significance i.e., associated with dysfunction/disease or vital to the protein’s function.^20−24^ These phosphorylation sites are S_366_,^21^ S_371_,^22^ S_376_,^22^ T_377_,^23^ S_378_,^22^ T_387_,^23^ and S_392_.^22,24^ The most extensively examined and highly conserved phosphorylation site, S_392_^24−26^ experiences significant phosphorylation levels during the G2 (the cell prepares for cell division by duplicating its genetic material and produces necessary proteins) and mitosis (cell division) stages of the cell cycle,^16,27^ and is also increased upon exposure to UV light and ionizing radiation.^28−30^ Additionally, some cancer-related p53 mutants exhibit increased phosphorylation on S_392_,^31^ and the phosphorylation on this site has been linked to modulation of p53 induced apoptosis.^32,33^ S_392_ stabilizes the tetrameric form of p53, which enables the DNA binding,^34^ and may also be involved in a liquid–liquid phase separation mechanism of p53.^35^ The stabilizing effect by PTMs can either be caused by the phosphorylation influence in the region, structural changes of the REG, or both. Structures derived from cryo-electron microscopy (CryoEM), see Figure 2b, suggest a particular interaction between REG and specific DBD residues. However, no dynamic investigation to date has observed this phenomenon. Our results highlight structural changes in the REG, which could indicate that the latter has a possible tetramer stabilizing effect; see Figure 2a.

Unfortunately, IDRs are frequently under-represented by researchers in favor of more stable and structured regions of proteins.^36^ Exploration of IDRs through biophysical techniques, such as nuclear magnetic resonance (NMR)^37^ and small-angle X-ray scattering (SAXS),^38^ yields significant insights into their dynamic properties, providing information on the average conformational state and the dynamics of IDRs. CryoEM^39^ is potentially useful, but limited to singular static structures and cannot capture the inherent dynamic nature of IDRs. Using physics-based force fields,^40^ molecular dynamics (MD) simulations provide valuable insight into IDRs^41,42^ accurately depending on the force field and the water model used. Simulations represent movements and interactions over time and allow experimental validation to determine protein states and dynamic behavior.^43^ Integrating these techniques with previous knowledge about the structure, design, and oligomers of p53, one can construct a complete understanding of the possible states of these regions. These intramolecular interactions can form important biochemical moieties that assist in facilitating the role of the protein.^44,45^

There is also growing attention to stabilizing elements within IDRs, such as induction of secondary structures^46^ and formation of salt bridges.^47,48^ Poly proline type II (PPII) helices play a crucial role in protein–protein and protein-nucleic acid interactions. PPII helices are also involved in various cellular processes such as transcription, cell motility, self-assembly, and elasticity.^49,50^ Despite its relevance, PPII helices are often neglected in protein structure determination and modeling due to their low presence in ordered proteins. However, regions with high quantities of Proline or disorder likely form PPII helix structures and can serve as recognition sites.^51,52^ Salt bridges, particularly those formed after PTMs, are also of significant interest, as phosphorylation observably increases the stability of the tetramer.^31^ Understanding the behavior of multiple IDRs requires understanding the factors, locations and processes that promote the formation of salt bridges.

A previous experimentally corroborated investigation has supported the restriction of mobility in the termini of an IDR as it dramatically affects its structural states.^13^ Specific IDRs act as dynamic linkers between two relatively static globular domains, such as the PTL, which connects the TET and the DBD. Terminally restricting these linkers enhances stability and provides a more accurate representation of the region. By inhibiting excessive fluctuations, we prevent disruptive vibrations or destabilizing events. The REG is a terminal IDR; thus, there is no defined restrained distance between the terminals. One might expect that the number of microstates achievable by an unrestrained trajectory and one restricted at one end would be identical. Although it is true that locking one terminal might not significantly affect the number of conformations permitted, it can still have a stabilizing effect on the IDR. Implementing such terminal restrictions on REG to test the influence of single-terminal restraint on dynamically evolving stabilizing features of the region would provide evidence that challenges the modern method by which we approach discrete IDR sections in mixed ordered/disordered proteins.

## Objectives

This study aims to comprehensively analyze the structural landscape of the disordered REG domain and the functional implications for p53. Moreover, we investigated the role of intramolecular interactions, particularly salt bridges involving Arginine residues, in stabilizing the REG. The impact of transient secondary structures, such as PPII helices, in stabilizing the region will be explored, and the effect of phosphorylation at S_392_ on the conformational dynamics and secondary structure of REG will be investigated. This will provide valuable information on the role of PTMs in the regulation of REG and its functional significance. Additionally, this study seeks to unravel the disruptions caused by ”over” phosphorylation in the REG and propose a potential explanation for the functional malfunctions associated with multisite phosphorylation. Our goal is to fully understand the influence of restricting one terminal of the REG on potential stabilizing intramolecular interactions.

## Methodology

All MD simulations were performed using the GROMACS package, version 2022.^53−56^ The AMBERSB99-ILDN force field, and a 4-point TIP4P-D^57^ water model were implemented, which were determined to be adequate in similar investigations on IDPs.^43^ While other MD techniques were considered (e.g., accelerated sampling techniques or replica exchange metadynamics), this investigation was meant as a proof of concept to establish some of the region’s behavior. This manuscript did not consider future investigations using these techniques or coarse-grained modeling, while potentially beneficial for future investigations. The starting structures were generated by fully extending residues 351–393 with Avogadro.^58^ Five residues of the tetramerization domain, 351–355, were incorporated to simulate the transition between the regions. The end terminals were simulated in zwitterionic form, bringing the net charge to +8. A rhombic box with a minimum distance of 10 nm from the residues was generated for the periodic boundary conditions, and eight chlorine ions were used to neutralize the charged residues. All Histidine residues were simulated at physiological pH with a neutral charge.

The leapfrog integrator with a time step of 2 fs was implemented with a neighbor search using the Verlet scheme using a grid algorithm with a 12 Å cutoff, and the electrostatic potential was implemented using the Ewald particle mesh method. The temperature coupling was performed with the Parrinello–Rahman barostat, and the Nose-Hoover thermostat maintained a temperature of 298 K. The LINCS algorithm was employed with hydrogen bond constraints. All simulations were minimized using the steepest descent algorithm and equilibrated at constant pressure, *NVT*, for 500 ps and constant volume, *NPT*, for one ns, where *N*, *T*, *V* and *P* are the number of particles, temperature, volume, and pressure, respectively. An additional 100 ns of relaxation time was run to allow the system to relax, but was not included in the analysis for each replicate. Five additional 1.1 μs trajectories were simulated with the N-terminal α-carbon restrained using the *freezegrps* command specified in the simulation mdp file, as previously described.^13^

**Figure 3:**
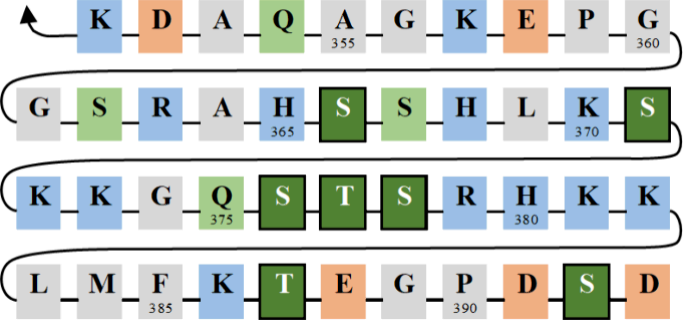
Characterization of the amino acid, AA, composition in the regulatory domain (REG), highlighting nonpolar in gray, polar in green, positively in blue, and negatively in red charged residues; biologically relevant phosphorylation sites indicated for reference shaded dark.

A control trajectory was simulated without modifications to the residues, hereafter referred to as REG_NP_, or REG_NP_^locked^ when restricted. To investigate the effects of phosphorylation, a trajectory, REG_SP_, was modulated with phosphates on the biologically relevant S_392_ residue, and another, REG_FP_, with all the phosphorylation sites observed experimentally. Phosphorylated residues (hereafter referred to as S1P for Serine and T1P for Threonine) alter the net charges of the REG_SP_ and REG_FP_ trajectories to +7 and +1, respectively. The corresponding number of ions was included to neutralize the charges. The specific AAs and their appearance in the sequence code are shown in Figure 3, with the phosphorylation sites highlighted. The sequence contains very few order-promoting residues (e.g., Tryptophan, Phenylalanine, Tyrosine) and several disorder-promoting residues (e.g., Serine or Lysine).^59^ The phosphorylation was accounted for using a modified force field by integrating the phosphorylated residue into the AMBER99SB-ILDN force field by adding new parameters for all the atoms, bonds, and impropers with charge, by modifying the AA rtp file and adding the new parameters to the *atomtypes* file. The new residue was also added to the AAs hdb file, and the TIP4P-D itp file was included to allow the use of the *-ignh* option. In addition, new parameters were added to the *ffnonbonded* file.

### Molecular Dynamics Analysis

The python package, *MdTraj*(60) was utilized to compute the root-mean-squared deviation, rmsd, to ensure that the trajectories were not trapped in local energy minima. The radius of gyration, R_G_, solvent-accessible surface area, SASA, and end-to-end distances, EE_DIST_, were also obtained from MdTraj to gain a better understanding of the overall size, shape, and conformation of the structures. Secondary structure predictions (DSSP) were produced using the GROMACS *gmx dssp* built-in command. SAXS scattering predictions were made using CRYSOL, an extension of the ATSAS package, and averaged to an ensemble of 50,000 frames (dt = 100 ps).^61^ The chemical shift, CS, predictor, Sparta+,^62^ was utilized to generate predictions to compare to experimental values. To assess the trajectories and systems’ ability to adapt and transition between states of the conformational landscapes, free-energy landscapes were produced by dimensional reduction using tICA^63^ and then processed by the PyEMMA built-in function.^64^

The python package, *SciPy*,^65^ was utilized for statistical and mathematical analysis, such as skew and kurtosis, to assess the data distribution.^66^ Skew is a measure of the asymmetry of a distribution, measuring the extent to which the data are skewed or stretched on one side. A positive skew means that the data have a long positive tail, whereas a negative skew indicates a long negative tail. The package *Sklearn*(67) was implemented for clustering using the agglomerative algorithm. The quality of the clusters was evaluated by calculating the silhouette score, which measures how well each data point matches its assigned cluster and ranges from −1 to 1 (higher scores indicate better intrinsic cluster agreement).^68^ The Python package *deeptime*(69) was implemented for the time-lagged independent component analysis, tICA, dimensionality reduction in the trajectories to generate an adequate conformational landscape. tICA is a variant of principal component analysis, PCA, although tICA is specifically designed to analyze time-dependent data such as MD trajectories, where there is a correlation between data points and time. The dihedral angles ϕ and ψ were used for the input fingerprints.

## Results and Discussion

### Conformational Dynamics and Flexibility

**Figure 4:**
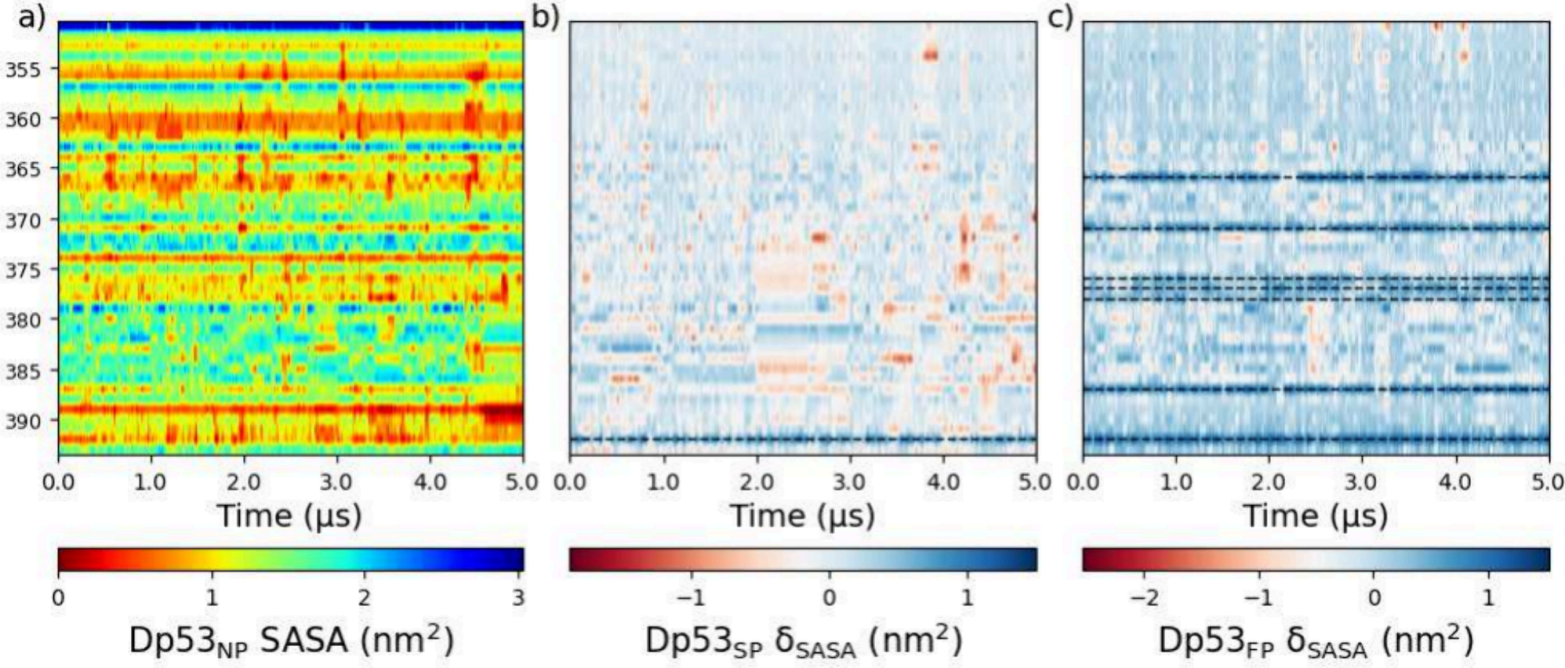
Total solvent-accessible surface area (SASA) computed by residue over time for the REG_NP_ (a), and the difference from the averages in (a) in REG_SP_ (b), and REG_FP_ (c). NP, SP, and FP represent nonphosphorylated, single-phosphorylated, and full-phosphorylated, respectively.

Analysis of RMSD plots (Figure S2) revealed that the trajectories did not exhibit trapping in any observable microstates or local minima. As seen in Table 1, the average EE_dist_ is slightly reduced in REG_SP_ and significantly reduced in REG_FP_. The distribution of EE_dist_ is also greater in REG_FP_ compared to REG_NP_ and REG_SP_, as evidenced by the values of the full-width half-maxima (fwhm) and variance (≈ 1.9 to 3.4 nm). This is further reflected by the change in R_G_, which is reduced by about 0.1 nm from REG_NP_ and REG_SP_ to REG_FP_. The higher positive skews in EE_dist_ and R_G_ indicate that the distributions are shifted and the more extended structures are further expanded from the mean than the contracted structures are compacted, as seen in their KDE plots (Figure S5). The total SASA by residue was computed to give insight into the structural dynamics and potential interactions with other molecules or intramolecularly. The average total SASA increased between REG_NP_/REG_SP_ and REG_FP_, although this does not necessarily suggest a more expanded state. This is most likely due to the increase in possible hydrogen bonding that multisite phosphorylation produces, with three available oxygen atoms and one protic hydrogen instead of one of each, and can be seen very pronounced in the phosphorylated residue (Figure 4). Since the actual residues were modified (Serine to Phosphoserine and Threonine to Phosphothreonine), a significant amount of the difference between the averages can be explained by the additional size and charges (Table S3).

**Table 1:** EE_DIST_, R_G_, and Total Solvent-Accessible Surface Area (SASA) Distributions Computed for Their Full-Width Half Maxima (FWHM), Skew, Kurtosis, and Respective Variance at Different Degrees of Phosphorylation

EE_DIST_	x̅ (nm)	fwhm (nm)	Skew	Kurtosis	Variance
REG_NP_^b^	3.614	3.319	0.352	–0.024	1.987
REG_SP_^b^	3.503	3.171	0.122	–0.444	1.814
REG_FP_^b^	3.123	4.333	0.793	0.050	3.386

R_G_	x̅ (nm)	fwhm (nm)	Skew	Kurtosis	Variance
REG_NP_^b^	1.738	0.797	0.637	0.067	0.114
REG_SP_^b^	1.719	0.692	0.559	–0.084	0.086
REG_FP_^b^	1.644	0.758	0.958	0.558	0.104

SASA^a^	x̅ (nm)	fwhm (nm)	Skew	Kurtosis	Variance
REG_NP_^b^	59.097	5.072	0.077	–0.445	4.639
REG_SP_^b^	58.099	6.494	0.064	–0.793	7.604
REG_FP_^b^	57.125	5.553	0.267	–0.153	5.559

a Total solvent-accessible surface areas (SASAs) only computed from nonphosphorylated residues.

b NP/SP/FP = nonphosphorylated, single-phosphorylated, and full-phosphorylated, respectively.

When the phosphorylation sites are controlled by removing them from the total SASA analysis (Table 1), a negative correlation is observed between the SASA and the number of PTMs. The effect is less pronounced if the phosphorylation sites are adjacent, such as S_376_, T_377_, and S_378_ (Figures S6 and S7). Notably, nonpolar residues (e.g., Alanine or Glycine) are not significantly affected by phosphorylation because (i) they are hydrophobic and prefer to be buried in the protein and not be exposed, and (ii) they contain no polar moieties (beyond the backbone) and play minor roles in many of the interactions associated with phosphorylated residues. As the first half of REG_351–393_ is largely nonpolar residues, the influence is much more pronounced toward the C-terminal. Two Arginine residues have SASAs which change significantly, as seen in Figure 4 and the SASA distribution/variances in Figure S7; R_379_ and R_363_. In R_379_, the variance in SASA is significantly reduced from 0.3 to 0.25 nm^2^ upon single-phosphorylation, and to 0.28 nm^2^ in overphosphorylation. This suggests that multisite phosphorylation in REG_351–393_ increases the potential for interactions with other molecules or intramolecularly primarily due to the formation of additional hydrogen bonds (Figure 4). The data also indicate that some residues, such as nonpolar residues and highly exposed Arginine residues, are less affected by phosphorylation, suggesting that they are not critical for the dynamic behavior of REG_351–393_.

#### Structural Insights

The secondary structure profile was determined from the trajectories using the built-in tool *dssp* in GROMACS (Table 2 and Figure S8) and plotted as time-dependent secondary structure plots in the SI (Figures S13 and S14). Single phosphorylation of REG results in a slight decrease in random coil, while full phosphorylation greatly increases the presence of disorders in the system. Additionally, the presence of α-/3_10_-helices is relatively unchanged upon phosphorylation; it is greatly disfavored in the overphosphorylated model. Interestingly, in the single-phosphorylated trajectory, a significant increase in β-sheets was detected from the nonphosphorylated trajectory (≈ 3%), a phenomenon which was either not observed or destroyed upon overphosphorylation. The results also demonstrate that the impact of phosphorylation is negatively correlated to the presence of PPII helices. Figures S9 - S12 show the percent instance of secondary structures by residues, and Figure S12 reveals that there is a strong presence of PPII helices in the region between S_367_ and G_374_, which is diminished upon overphosphorylation. The β-sheets/bridges which were favored in REG_SP_ were also highly localized with residue regions K_374_ - S_376_ and M_384_ - E_388_, as seen in Figure S10.

**Table 2:** Secondary Structure Predictions (%) for Each of the Trajectories Using DSSP Implemented by GROMACS

Trajectory^a^	Coils	Bridges^b^	Helices^b^	PPII	Bend	Turn	Phos.
REG_NP_	56.9	1.39	1.61	8.62	22.2	9.27	0.00
REG_SP_	55.0	3.07	1.87	7.57	21.0	9.21	2.33
REG_FP_	66.3	0.48	0.29	4.68	8.41	3.54	16.3

b , combination of isolated and extended β-sheets and (c) a combination of 3,4, and 5-turn α-helices.

a , NP/SP/FP = nonphosphorylated, single-phosphorylated, and full-phosphorylated, respectively.

**Figure 5:**
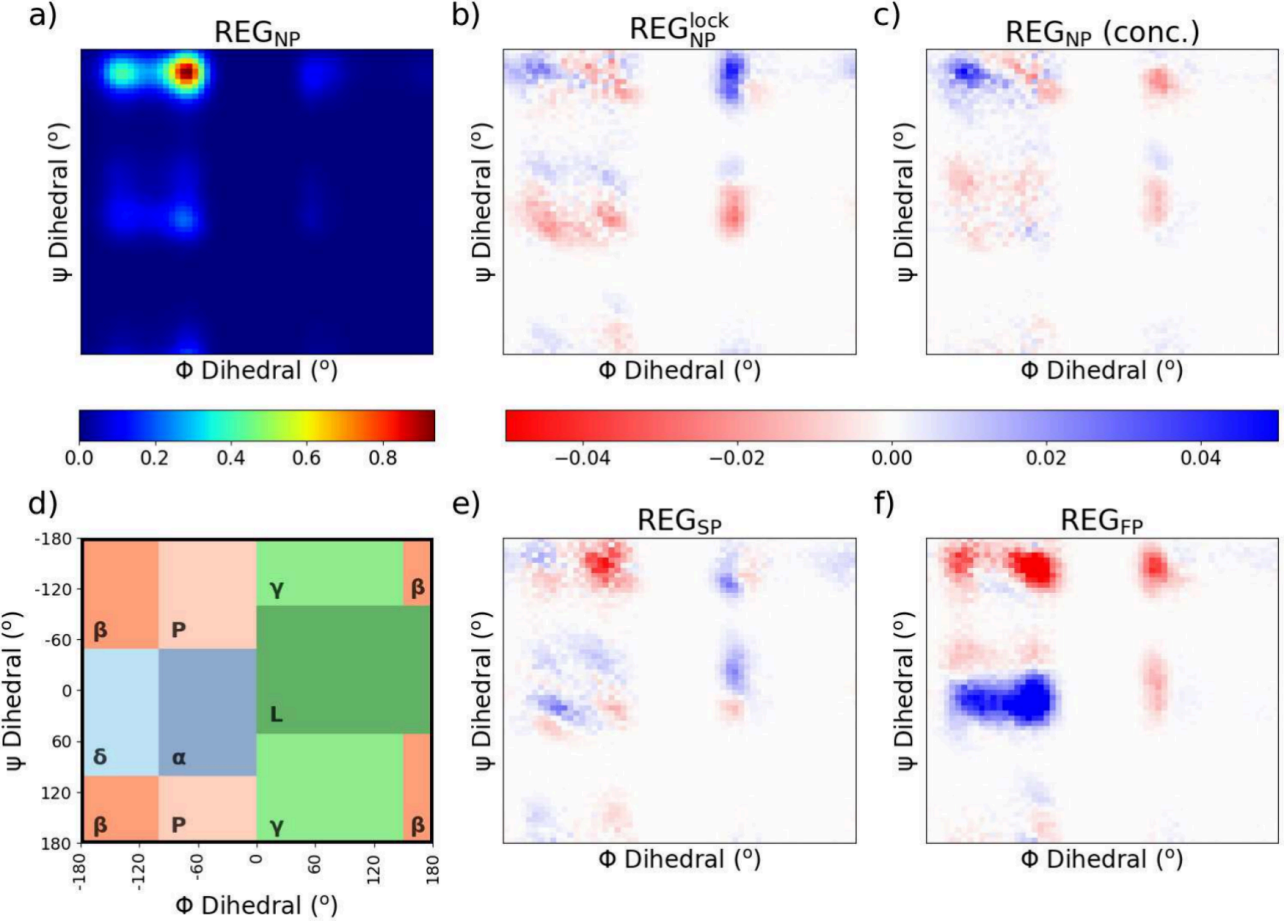
Integrated Ramachandran plots from (a) the nonphosphorylated REG_NP_ as well as the change in the (b) restrained, REG_NP_^lock^, (c) concentrated, REG_NP_^lock^, (e) single-phosphorylated, REG_SP_, and (f) fully phosphorylated, REG_FP_ trajectories. Regions associated with commonly expressed secondary structures (d) are plotted as a reference (α: normal α-helix region; δ: deformed α-helix region; β: β-sheet region; P: PPII helix region; L: left-handed helix; γ: ϕ > 0°).

We also investigated the change in the distribution of the dihedral angles in an integrated Ramachandran plot (Figure 5), in which each of the ϕ and ψ angles was plotted into regions and the difference between the populations of the areas after phosphorylation was analyzed. Except for Glycine, Proline, or Proline-adjacent residues, most AAs can be generalized into specific regions that indicate a preference for secondary structure (Figure 5d).^70,71^ There are a considerable number of residues residing in the region ϕ = [-180°, −120°] and ψ = [-180°, 60°], associated with β-sheets of PPII helices. These residues are substantially diminished upon single and full phosphorylation, and instead replaced by dihedrals in the region ϕ = [-180°, −120°] and ψ = [-60°, 60°]. This transition is highly localized in REG_FP_ but widely dispersed in REG_SP_, indicating that the structures in the single-phosphorylated trajectory are more varied. Specific changes in angular distribution profile changes can be described in the Supporting Information (Figures S15 - S26). The analysis of specific residue dynamics upon phosphorylation of the region reveals interesting insights into the effect of phosphorylation on protein structure and function.

It is observed that the phosphorylation state of specific residues, specifically R_363_, K_373_, H_380_, L_383_, K_386_, and T_387_, can significantly impact their dynamics (Figures S16 S20). These residues show a significant shift in their angle distributions upon single phosphorylation, indicating a conformational change induced by phosphorylation. However, this change is diminished or eliminated upon overphosphorylation, suggesting that overphosphorylation may have a stabilizing/destabilizing effect on these residues. In contrast, residues E_358_ and D_391_ show a complete elimination of their angle distributions upon both single and overphosphorylation (Figure S15), indicating that phosphorylation, regardless of the position, induces a global conformational change in the protein structure. Furthermore, the analysis also reveals that the effect of phosphorylation on residue dynamics is not uniform across all residues. For example, residues K_381_, F_385_, E_388_, K_386_, T_377_, and S_378_ only show changes in their angle distributions when the protein is overphosphorylated (Figures S21 -S26). This suggests that these residues may have a higher threshold for phosphorylation-induced conformational changes, which could potentially affect the behavior of the REG.

### Protein Interactivity

**Figure 6:**
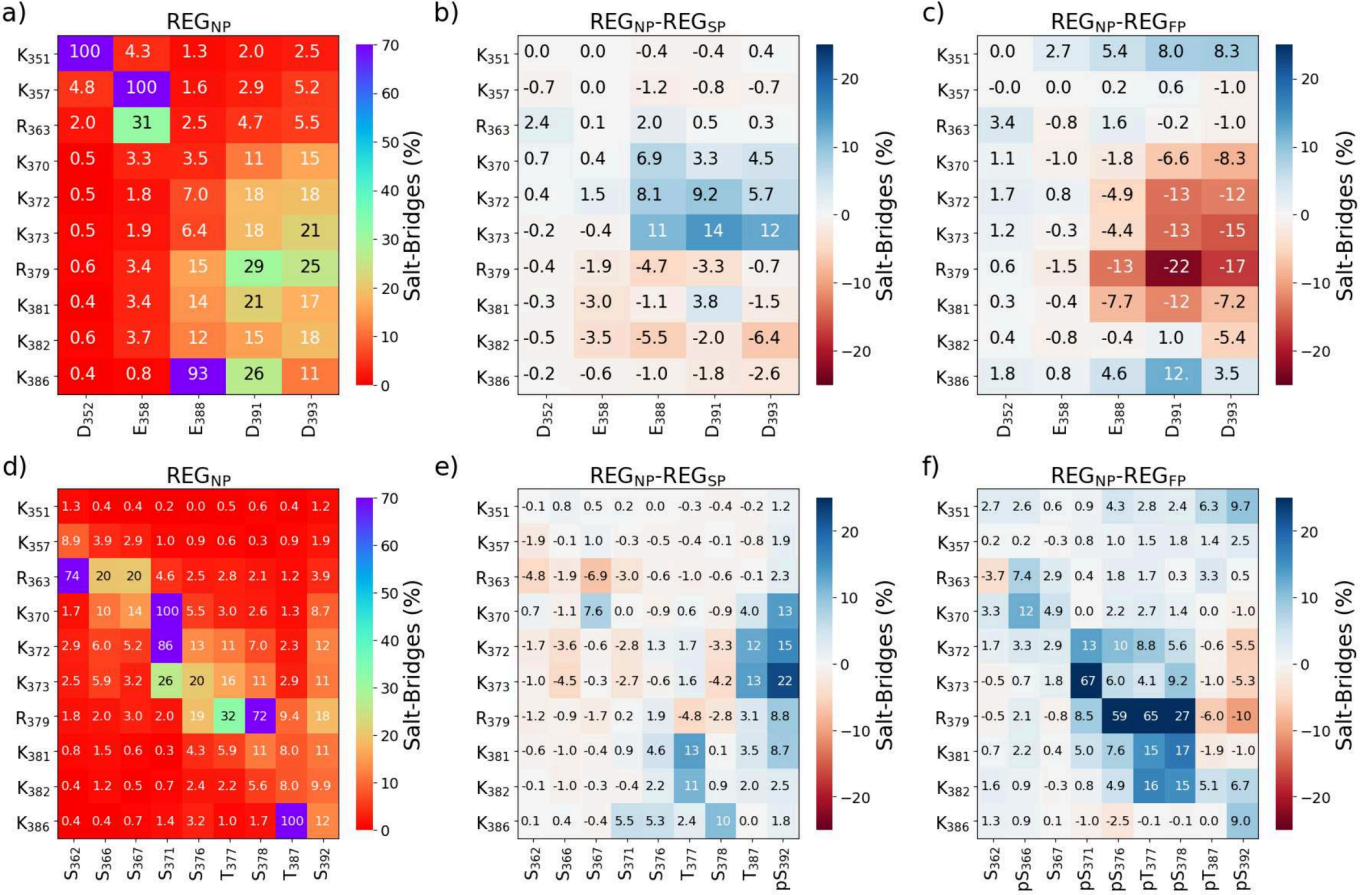
Heatmaps demonstrating (a) the instances of salt bridges forming between charged species in the protein, as well as changes upon (b) single phosphorylation and (c) “over” phosphorylation. Also included is (d) the propensity for salt bridges/hydrogen bonding to form in the protein and the influence on these interactions by (e) single phosphorylation and (f) “over” phosphorylation. A salt bridge/hydrogen bond is defined as the atoms are within 0.3 nm distance of each other.

A sizable amount of research points to the relevance of ionic or electrostatic bonds (salt bridges) as contributing factors in the stabilization and function of IDRs.^47,72,73^ These bonds form between charged species in the protein chain; 15 (+5 net charge) in REG_NP_, 16 (+4) in REG_SP_, and 22 (−2) in REG_FP_. The phosphorylation at S_392_ slightly increases the average occurrences of salt bridges, where a salt bridge is observed when the negative and positive species are within 3.5 Å, from 3.87% to 4.05%. The trend is reversed in REG_FP_, which decreases to 3.70%, which is explainable by increased charged residues by phosphorylation, although overphosphorylation indicates a notable decrease in salt bridges. Most of these bridges are restricted to local interactions closer to the tetramerization domain (Figure 6a-c), such as K_351_ ··· D_352_ (42%), K_357_ ··· E_358_ (39%), or K_386_ ··· E_388_ (31%) near the C-terminus. These interactions, being local, are not significantly influenced by singular phosphorylation at S_392_, although salt bridges between K_373_ and D_391_/D_393_ increase by 7.1% and 6.7% respectively. Furthermore, salt bridges are significantly reduced by 5.8%, and 7.6%, respectively, upon overphosphorylation. Other interactions are similarly disfavored in REG_FP_, such as nearly all R_379_ bridges, most notably R_379_ ··· D_391_ (−12%), or R_379_ ··· D_393_ (−9%). Instead, salt bridges in REG_FP_ prefer local interactions, with an increase between K_386_ ··· E_388_ (11%) and K_386_ ··· D_391_ (5.8%) or a slight increase between end-terminals.

Upon phosphorylation, newly charged species can form salt bridges across the segment. Phosphorylation at site S_392_, for example (Figure 6d-f), results in substantially increased salt bridges between positively charged residues R_363_ and K_381_ (10–20%). These interactions are not enforced upon overphosphorylation, and hydrogen bonding between the residues is discouraged in the region. Instead, there is a preference for local (within ten residues) or terminal interactions (N to C). Furthermore, the trajectory has a strong preference for local interactions in REG_FP_, not seen in the other two trajectories, such as R_379_ ··· pT_377_ (97%) or R_379_ ··· pS_376_ (77%), as can be seen in Figure 6 in the Supporting Information. These interactions of R_379_ detract from the natural salt bridges formed in REG_NP_, and local interactions also prohibit the formation of traditional stable secondary structures in the regions. This influence is perhaps most evident in the bivariate density plots (Figure S27), where it can be seen that an ensemble produced of conformations where R_379_ is bound with S_392_, has different influences on R_G_ depending on the level of phosphorylation. If the two residues form a salt bridge in REG_NP_, the R_G_ increases slightly. This trend is reversed when the salt bridge forms in both REG_SP_ and REG_FP_.

### Energy Landscapes and Conformational Ensembles

**Figure 7:**
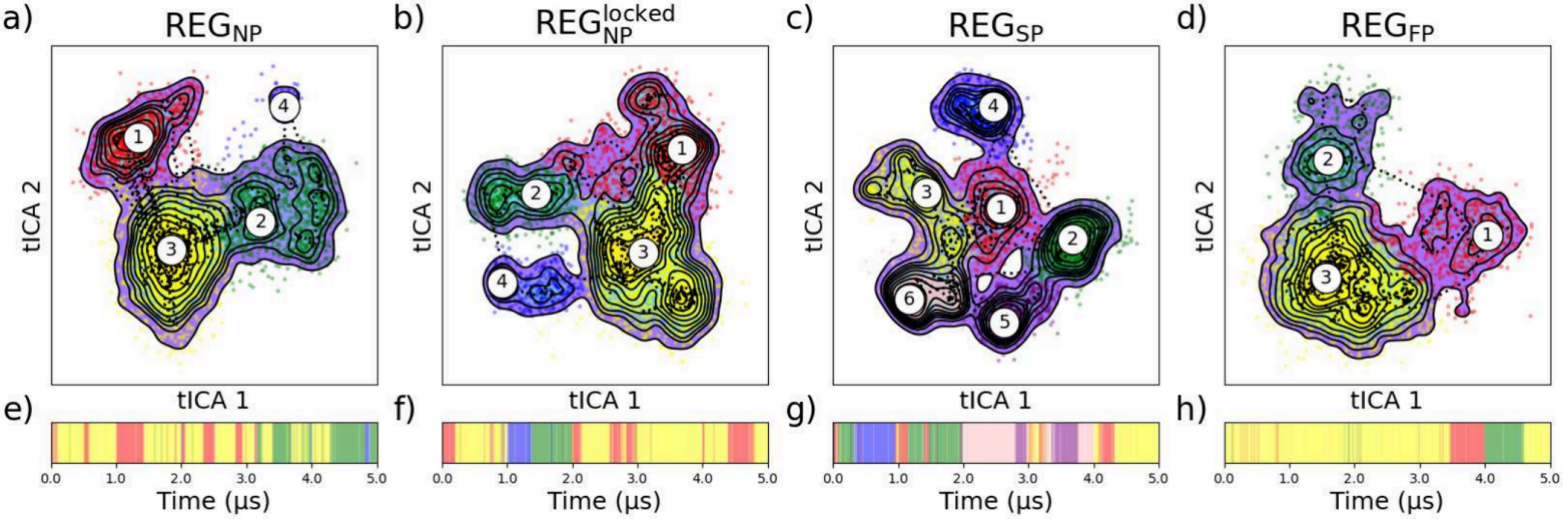
Free energy plots generated using tICA dimensionality reduction on ϕ and ψ angles, with centers identified by agglomerative hierarchical clustering and Gaussian KDE for each of the trajectories (a-d). Rolling averages are plotted (dotted) to represent the path that the trajectories take among the states, and the cluster assignment by time is displayed (e-i) below. NP, SP, and FP represent nonphosphorylated, single-phosphorylated, and full-phosphorylated, respectively.

To categorize the different states achievable in differently phosphorylated systems of REG, we utilized the linear dimensionality reduction algorithm tICA to generate four latent spaces from the different trajectories (Figure 7). Agglomerative clustering determined several high-density conformations in these clusters, as determined from several internal clustering evaluation metrics such as silhouette score (SS) and Davies Bouldin index (DB), as seen in the Supporting Information (Figure S29). The best-performing clustering sizes determined from the silhouette score were REG_NP_ (3), REG_NP_^lock^ (3), REG_SP_ (6), and REG_FP_ (3). Similar ideal cluster sizes were determined from the Davies Bouldin index; REG_NP_ (5), REG_NP_^lock^ (4), REG_SP_ (7), and REG_FP_ (4), although some of these clusters were sparsely populated (<10 conformations) and were omitted from the analysis. A notable observation lies in the divergent expansion of discernible conformational states within the REG_SP_ system relative to the nonphosphorylated entities (i.e., REG_NP_ and REG_NP_^locked^) and the excessively phosphorylated segment (REG_FP_).

**Figure 8:**
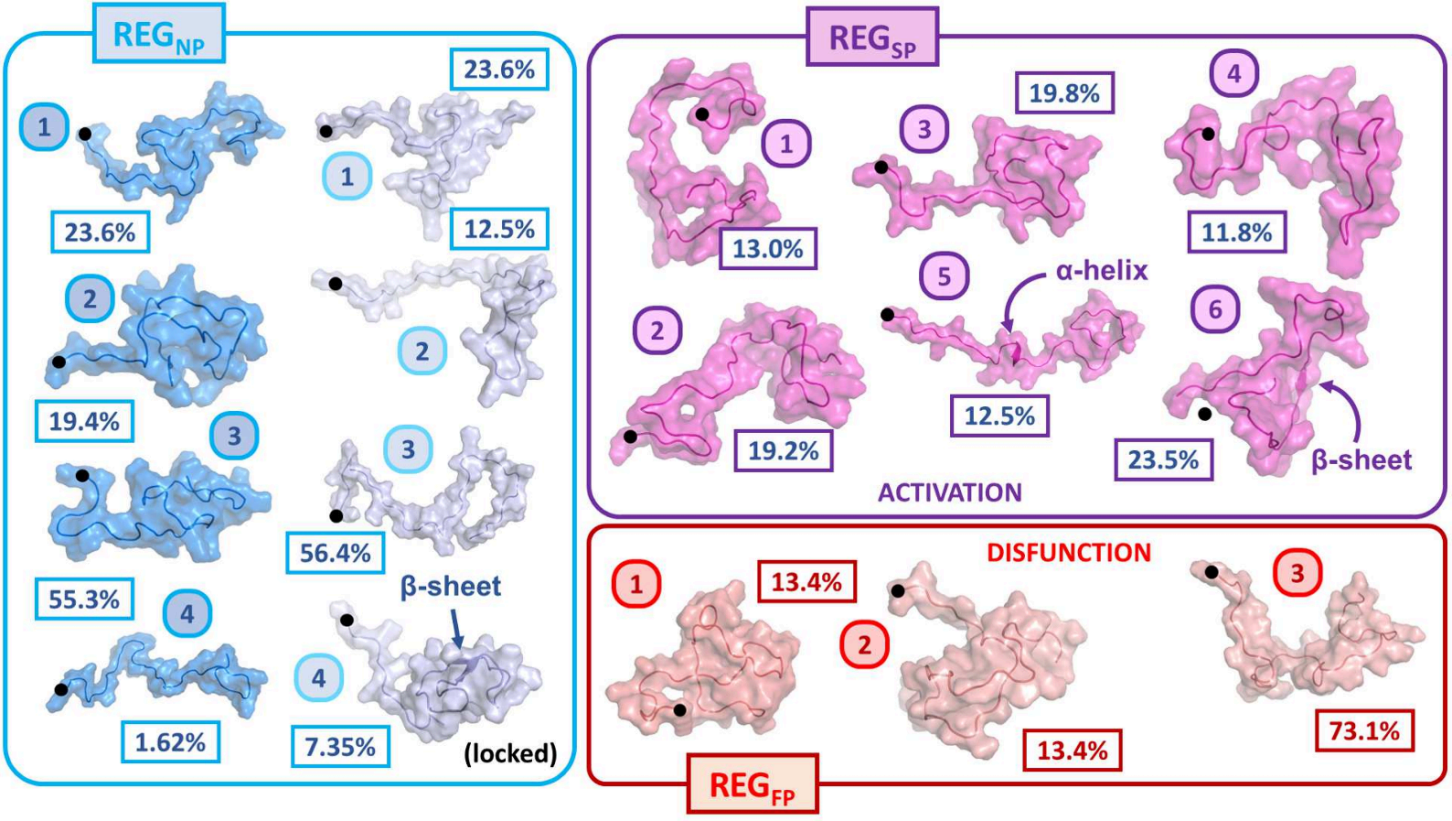
Representative abstract structures from the four REG_NP_, four REG_NP_^lock^, six REG_SP_, and three REG_FP_ cluster centers as well as their relative percent occurrence throughout the trajectories. Black markers are included to indicate the N terminus, and specific secondary structures are highlighted.

More in-depth analysis of the different states achieved by this clustering can be seen as described in the SI, describing the globular trends among each cluster (Table S4 and Figure S28) as well as the secondary structure characteristics of each ensemble (Table S5). In the phosphorylated trajectories, there is a profound difference between the states achievable in REG_SP_ and REG_FP_, both in the variance of the conformational landscape and the specific structures produced. REG_SP_[5], comprising 12.5% of the trajectory, contains a significant presence of α-helix, unique to the single phosphorylated trajectory. Also of note, REG_SP_[6], spanning 23.5% of the trajectory, has a preponderance of β-sheets, similar to REG_NP_^lock^[4]. REG_FP_ produces a landscape that is significantly less diverse, with only three states detected states; one that embodies the majority (73. 1%) of the trajectory (REG_FP_[3]) more expanded (>3 nm), and two states (REG_FP_[3] and REG_FP_[2]) with a smaller EE_DIST_ (<3 nm). The landscapes suggest the existence of additional conformational states achievable in REG_SP_, which are diminished upon ”over”-phosphorylation. Not only are the different conformational states different among different phosphorylation levels, but the distinction between the groups and how they are distributed on the derived latent space is altered. In REG_SP_, the conformational states are tightly clustered together and resemble separate islands. This might indicate that the intermediary conformations between states have high energy levels and that transitions are not preferred. In the REG_FP_ landscape, the barriers between the clusters are less pronounced, suggesting a smoother transition between states. Representatives from each of these states can be seen visually for each of the trajectories in Figure 8.

### Influence of N-Terminal Restraint

As described in the methodology, a modulation of the trajectory was performed, restricting the movement of the N-terminal residue that would be connected to the rest of the p53 protein. There were several notable and interesting results from the modulated trajectory. The influence of terminal restraint (REG_NP_^lock^) on globular properties shows a slight increase in EE_DIST_ and R_G_ (Table S1) and a significant decrease in total SASA from the free trajectory, REG_NP_. In contrast to what was expected, restricting one terminal of the protein fragment actually increased the variation in each of the three global properties investigated. In terms of secondary structures, there was no significant difference between REG_NP_ and REG_NP_^lock^, with a notable exception seen in Figures S8 and S13 in which the instances of β-sheets were significantly more pronounced. Between the nonphosphorylated trajectories, terminally restraining the trajectory has a small but significant influence on the states achievable in REG. In particular, on both trajectories, the largest identified states were REG_NP_[3] and REG_NP_^lock^[3], which encompasses ≈55% of both trajectories. Both clusters exhibit a tendency toward random coils and an average or below-average propensity for other secondary structures compared to the complete trajectory’s average. Restraining the trajectory replaces a minor 1.6% state (REG_NP_[4]), with a high affinity for PPII helices with a more significant (7.35%) state (REG_NP_^lock^[4]) showing a substantial β-sheet presence. This slight modulation of the conformational landscape suggests that the restraint of the terminal IDR plays a minor role in the stabilization of transient secondary structures. The results show that the method of restricting motion in a single terminal of a trajectory has an influence on the possible structures that can be produced, as visualized in Figure 7b, even in IDRs that are terminal.

### Influence of Salt Concentration

Finally, the control trajectory, REG_NP_ was also simulated using a concentration of 100 mM ionic solvent to test solvent effects. This modulation of the trajectory created the most drastic observed influence on the global properties of the REG, increasing the EE_dist_ and greatly increasing the R_G_ and SASA (Table S1). Additionally, the variance in these values was greatly heightened, indicating a more stochastic and free-moving structure. The secondary structures (Figure S8) showed no significant change, with an overall decrease in structure and an increase in random coils. Salt bridges were disfavored, preventing partial intramolecular stabilization from charged species within REG_NP_^conc.^. Based on these findings, it is recommended that further research be conducted on the impact of salt concentration on the organization of IDPs in order to verify their accuracy using empirical methods.

### Experimental Validation

The accuracy of this investigation outcome is heavily dependent on the reliability and accuracy of the applied force fields, as well as the specific parameters used in the MD simulations. Chemical shift predictions were conducted on each trajectory using Sparta+, and the results were subsequently cross-checked against available chemical shift data^74^ to determine accuracy. The precision of the ^1^5N chemical shift predictions is high, indicated by a Pearson coefficient of 0.937 when compared to experimental data (Figure S30a). The ^1^H_N_ CSs are more difficult to predict due to a smaller range for shielding values, as well as mixed environmental, local influences and hydrogen bonding. Nevertheless, the r^2^ values were improved from 0.178 in REG_NP_ to 0.218 in REG_NP_^lock^, indicating the value of terminal restriction for agreement with experimental data. Specific changes upon phosphorylation were not tracked due to a lack of phosphorylated NMR CS data, as well as an inability for Sparta+ to account for phosphorylation, although the CS predictions and their changes were plotted as a function of phosphorylation in the Supporting Information for future reference, should more experimental data become available (Figure S31).

In addition to comparing the NMR chemical shifts, several crystallized structures were used as references to understand the findings of this investigation. In its monomeric form (8f2i),^75^ the EE_DIST_ is approximately 2.37 nm in crystallized state. The missing information from the CryoEM structure of the tetramer means that the terminal EE_DIST_ of REG_351–393_ is currently unavailable. However, there is information on many residue positions in two REG_351–393_ strands. The distance between the α-carbons in the tetrameric form of K_351_ and K_386_ is 4.62 nm, much longer than that observed in the crystallized monomeric form. These identical residues are 1.56 nm apart in monomeric form. In the different trajectories, they are 3.96 ± 1.52, 3.97 ± 1.35, and 3.19 ± 1.51 nm in the REG_NP_, REG_SP_, and REG_FP_ trajectories, respectively, suggesting that influences of the overphosphorylation constrain the region to a smaller span, further from the observed tetramerized span, possibly given evidence to its dysfunction. Based on the CRYSOL SAXS profiles (Figure S1a), the REG_SP_ and REG_NP_ are similar in their scattering plots; however, REG_FP_ produces different structures and presents far more disorder or disorder promoting behavior.

## Conclusion

This study delved into the influence of phosphorylation on the structural dynamics and possible functional behavior of REG in p53. While single phosphorylated at S_392_ (REG_SP_) had a negligible effect on R_G_ and a subtle decrease in EE_dist_, multisite phosphorylation (REG_FP_) significantly decreases both. Interestingly, based on experimental models, the distance between K_351_ and K_386_ in the REG_NP_ and REG_SP_ trajectories closely resembles the tetrameric form of p53, while in contrast, the REG_FP_ trajectory more closely resembles the monomeric form. The NMR CS predicted by Sparta+ shows a good agreement with the experimental data (r^2^ = 0.937), partially validating the conformational ensembles generated from the trajectories. The influence of SASA was investigated in two terms, including phosphorylated residues and excluding them. The first was included to present the differences between the structures as different proteins, and the second was included to assess the influence of phosphorylation on the other residues. The total SASA decreased slightly in REG_SP_ and increased significantly in REG_FP_. We find a negative correlation between PTMs and SASA upon controlling for the phosphorylated residues. The phosphorylation of REG at site S_392_ results in a substantial increase in the salt bridges of R_363_ and K_381_ (10–20%). This effect is not observed in multisite phosphorylation, where hydrogen bonding and salt bridges are discouraged in the region. Instead, REG_FP_ shows a preference for local (within 10 residues) or terminal interactions (N to C).

Single phosphorylation induces significant changes at specific residues, such as R_363_, K_373_, H_380_, L_383_, K_386_, and T_387_ in their angle distributions, however, this effect appears to be diminished or even eliminated when the protein is overphosphorylated, indicating that excessive phosphorylation might destabilize these phenomena. In contrast, residues such as E_358_ and D_391_ completely eliminate their angle distributions upon both single and overphosphorylation, pointing to a global conformational change within the protein structure induced by phosphorylation at any level. Furthermore, certain residues, including K_381_, F385, E_388_, K_386_, T_377_, and S_378_, only show changes in their angle distributions when the protein is overphosphorylated, suggesting a higher threshold for phosphorylation-induced conformational changes in these specific residues. This indicates that the impact of phosphorylation on residue dynamics is not uniform across the REG domain and that different residues may respond differently to phosphorylation, potentially affecting the behavior and function of the REG domain in various ways. While these phenomena could provide an explanation for the experimental observations on the dysfunction of overphosphorylated p53 in the REG, it warrants future site-specific investigations with NMR at different phosphorylated levels to confirm this.

Single phosphorylation has been observed to partially reduce PPII helices, while overphosphorylation increases random coil formation, indicating a shift toward more disordered structures. Phosphorylation leads to a profound difference in the states that can be achieved in REG, both in terms of the variance of the conformational landscapes and the specific structures produced. In the single phosphorylated simulation, a unique state shows a minor but significant transient structure of α-helices, which was unique to this phosphorylation level and was observed in multiple replicates. Another state observed in REG_SP_ was one that contained a preponderance for β-sheets. These additional accessible conformational states are suggested to add some level of functionality to the protein, all of which are diminished or removed in overphosphorylated simulations.

Research delineates how phosphorylation at specific sites, such as S_392_, influences not only binding affinities and behaviors but also induces notable alterations in secondary structures, SASA, and salt bridge formation. The distinction between single and overphosphorylation is critical, revealing that while the former can introduce beneficial structural features and potentially enhance protein functionality, the latter may lead to significant dysfunction by disrupting the protein’s conformational landscape and stabilizing interactions. However, the study is not without limitations, including the inherent challenges of accurately simulating the dynamic nature of intrinsically disordered proteins and the potential for oversimplification of complex in vivo phosphorylation dynamics. Future research could benefit from integrating experimental data to guide computational models and explore the effects of phosphorylation in a broader range of cellular contexts.

## Supporting Information Available

Additional data and analysis for our study on the effects of phosphorylation levels on the conformational dynamics of REG. We compared trajectories with varying levels of phosphorylation and assessed their consistency and quality through Kratky plots. The time-dependent behavior of the root-mean-square deviation (RMSD), the radius of gyration (R_G_), and the end-to-end distance (EE_DIST_) were also compared to explore the flexibility changes of REG. We analyzed changes in protein phosphorylation and total solvent-accessible surface area (SASA) using KDE plots. Furthermore, we examine changes in the dihedral angle distribution, variation of SASA by residue, and P-values for R_G_, EE_dist_, and total SASA between trajectories for statistical analysis. DSSP plots provide a detailed assessment of structural heterogeneity, while salt-bridge and hydrogen-bonding heat maps analyze interactions and formation in different trajectories. We generated Ramachandran plots for residues significantly impacted by phosphorylation and compared the salt bridge and hydrogen bonding betweenS_392_ and other charged residues. Lastly, tICA analysis was performed on each trajectory to describe the conformational landscape of REG_351–393_ and identify changes after phosphorylation (PDF)

## References

[ref1] Oren M. Regulation of the p53 tumor suppressor protein. J. Biol. Chem. 1999, 274, 36031–36034. 10.1074/jbc.274.51.36031. 10593882

[ref2] Vousden K. H. Activation of the p53 tumor suppressor protein. Biochimica et Biophysica Acta (BBA)-Reviews on Cancer 2002, 1602, 47–59. 10.1016/S0304-419X(02)00035-5. 11960694

[ref3] Maximov G.; Maximov K. The role of p53 tumor-suppressor protein in apoptosis and cancerogenesis. Biotechnology & Biotechnological Equipment 2008, 22, 664–668. 10.1080/13102818.2008.10817532.

[ref4] Madden S. L.; Galella E. A.; Riley D.; Bertelsen A. H.; Beaudry G. A. Induction of cell growth regulatory genes by p53. Cancer research 1996, 56, 5384–5390. 8968090

[ref5] Sionov R. V.; Hayon I. L.; Haupt Y. Madame Curie Bioscience Database [Internet]; Landes Bioscience, 2013.

[ref6] Sullivan K. D.; Galbraith M. D.; Andrysik Z.; Espinosa J. M. Mechanisms of transcriptional regulation by p53. Cell Death & Differentiation 2018, 25, 133–143. 10.1038/cdd.2017.174. 29125602 PMC5729533

[ref7] Molchadsky A.; Rivlin N.; Brosh R.; Rotter V.; Sarig R. p53 is balancing development, differentiation and de-differentiation to assure cancer prevention. Carcinogenesis 2010, 31, 1501–1508. 10.1093/carcin/bgq101. 20504879

[ref8] Carson D. A.; Lois A. Cancer progression and p53. Lancet 1995, 346, 1009–1011. 10.1016/S0140-6736(95)91693-8. 7475551

[ref9] Wang Y.; Reed M.; Wang P.; Stenger J. E.; Mayr G.; Anderson M. E.; Schwedes J. F.; Tegtmeyer P. p53 domains: identification and characterization of two autonomous DNA-binding regions. Genes & development 1993, 7, 2575–2586. 10.1101/gad.7.12b.2575. 8276240

[ref10] Raj N.; Attardi L. D. The transactivation domains of the p53 protein. Cold Spring Harbor perspectives in medicine 2017, 7, a026047 10.1101/cshperspect.a026047. 27864306 PMC5204331

[ref11] Pavletich N. P.; Chambers K. A.; Pabo C. O. others The DNA-binding domain of p53 contains the four conserved regions and the major mutation hot spots. Genes and development 1993, 7, 2556–2564. 10.1101/gad.7.12b.2556. 8276238

[ref12] Xue B.; Brown C. J.; Dunker A. K.; Uversky V. N. Intrinsically disordered regions of p53 family are highly diversified in evolution. Biochimica et Biophysica Acta (BBA)-Proteins and Proteomics 2013, 1834, 725–738. 10.1016/j.bbapap.2013.01.012. 23352836 PMC3905691

[ref13] Bakker M. J.; So̷rensen H. V.; Skepo M. Exploring the Role of Globular Domain Locations on an Intrinsically Disordered Region of p53: A Molecular Dynamics Investigation. J. Chem. Theory Comput. 2024, 20, 1423–1433. 10.1021/acs.jctc.3c00971. 38230670 PMC10867847

[ref14] Kim H.; Kim K.; Choi J.; Heo K.; Baek H. J.; Roeder R. G.; An W. p53 requires an intact C-terminal domain for DNA binding and transactivation. Journal of molecular biology 2012, 415, 843–854. 10.1016/j.jmb.2011.12.001. 22178617 PMC3267882

[ref15] Terakawa T.; Takada S. p53 dynamics upon response element recognition explored by molecular simulations. Sci. Rep. 2015, 5, 17107 10.1038/srep17107. 26596470 PMC4656996

[ref16] Poyurovsky M. V.; Katz C.; Laptenko O.; Beckerman R.; Lokshin M.; Ahn J.; Byeon I.-J. L.; Gabizon R.; Mattia M.; Zupnick A.; et al. The C terminus of p53 binds the N-terminal domain of MDM2. Nature structural & molecular biology 2010, 17, 982–989. 10.1038/nsmb.1872. PMC2922928 20639885

[ref17] Gu B.; Zhu W.-G. Surf the post-translational modification network of p53 regulation. International journal of biological sciences 2012, 8, 672 10.7150/ijbs.4283. 22606048 PMC3354625

[ref18] DeHart C. J.; Chahal J. S.; Flint S.; Perlman D. H. Extensive post-translational modification of active and inactivated forms of endogenous p53. Molecular & Cellular Proteomics 2014, 13, 1–17. 10.1074/mcp.M113.030254. 24056736 PMC3879606

[ref19] Appella E.; Anderson C. W. Post-translational modifications and activation of p53 by genotoxic stresses. European journal of biochemistry 2001, 268, 2764–2772. 10.1046/j.1432-1327.2001.02225.x. 11358490

[ref20] Yakovleva T.; Pramanik A.; Kawasaki T.; Tan-No K.; Gileva I.; Lindegren H.; Langel U.; Ekstrom T. J.; Rigler R.; Terenius L.; et al. p53 latency: C-terminal domain prevents binding of p53 core to target but not to nonspecific DNA sequences. J. Biol. Chem. 2001, 276, 15650–15658. 10.1074/jbc.M100482200. 11279079

[ref21] Park J. H.; Smith R. J.; Shieh S.-Y.; Roeder R. G. The GAS41-PP2Cβ complex dephosphorylates p53 at serine 366 and regulates its stability. J. Biol. Chem. 2011, 286, 10911–10917. 10.1074/jbc.C110.210211. 21317290 PMC3064146

[ref22] Ashcroft M.; Kubbutat M. H.; Vousden K. H. Regulation of p53 function and stability by phosphorylation. Molecular and cellular biology 1999, 19, 1751–1758. 10.1128/MCB.19.3.1751. 10022862 PMC83968

[ref23] Yogosawa S.; Yoshida K. Tumor suppressive role for kinases phosphorylating p53 in DNA damage-induced apoptosis. Cancer science 2018, 109, 3376–3382. 10.1111/cas.13792. 30191640 PMC6215896

[ref24] Cox M. L.; Meek D. W. Phosphorylation of serine 392 in p53 is a common and integral event during p53 induction by diverse stimuli. Cellular signalling 2010, 22, 564–571. 10.1016/j.cellsig.2009.11.014. 19932175

[ref25] Maclaine N. J.; Hupp T. R. The regulation of p53 by phosphorylation: a model for how distinct signals integrate into the p53 pathway. Aging 2009, 1, 490 10.18632/aging.100047. 20157532 PMC2806026

[ref26] Bode A. M.; Dong Z. Post-translational modification of p53 in tumorigenesis. Nature Reviews Cancer 2004, 4, 793–805. 10.1038/nrc1455. 15510160

[ref27] Buschmann T.; Adler V.; Matusevich E.; Fuchs S. Y.; Ronai Z. p53 phosphorylation and association with murine double minute 2, c-Jun NH2-terminal kinase, p14ARF, and p300/CBP during the cell cycle and after exposure to ultraviolet irradiation. Cancer Res. 2000, 60, 896–900. 10706102

[ref28] Blaydes J. P.; Hupp T. R. DNA damage triggers DRB-resistant phosphorylation of human p53 at the CK2 site. Oncogene 1998, 17, 1045–1052. 10.1038/sj.onc.1202014. 9747884

[ref29] Wallace M.; Coates P.; Wright E.; Ball K. Differential post-translational modification of the tumour suppressor proteins Rb and p53 modulate the rates of radiation-induced apoptosis in vivo. Oncogene 2001, 20, 3597–3608. 10.1038/sj.onc.1204496. 11439323

[ref30] Finlan L. E.; Nenutil R.; Ibbotson S. H.; Vojtesek B.; Hupp T. R. CK2-site phosphorylation of p53 is induced in ΔNp63 expressing basal stem cells in UVB irradiated human skin. Cell Cycle 2006, 5, 2489–2494. 10.4161/cc.5.21.3393. 17106255

[ref31] Furihata M.; Kurabayashl A.; Matsumoto M.; Sonobe H.; Ohtsuki Y.; Terao N.; Kuwahara M.; Shuin T. Frequent phosphorylation at serine 392 in overexpressed p53 protein due to missense mutation in carcinoma of the urinary tract. Journal of Pathology 2002, 197, 82–88. 10.1002/path.1082. 12081208

[ref32] Yap D. B.; Hsieh J.-K.; Zhong S.; Heath V.; Gusterson B.; Crook T.; Lu X. Ser392 phosphorylation regulates the oncogenic function of mutant p53. Cancer research 2004, 64, 4749–4754. 10.1158/0008-5472.CAN-1305-2. 15256442

[ref33] Castrogiovanni C.; Waterschoot B.; De Backer O.; Dumont P. Serine 392 phosphorylation modulates p53 mitochondrial translocation and transcription-independent apoptosis. Cell Death & Differentiation 2018, 25, 190–203. 10.1038/cdd.2017.143. 28937686 PMC5729520

[ref34] Sakaguchi K.; Sakamoto H.; Lewis M. S.; Anderson C. W.; Erickson J. W.; Appella E.; Xie D. Phosphorylation of serine 392 stabilizes the tetramer formation of tumor suppressor protein p53. Biochemistry 1997, 36, 10117–10124. 10.1021/bi970759w. 9254608

[ref35] Dai Z.; Li G.; Chen Q.; Yang X. Ser392 phosphorylation modulated a switch between p53 and transcriptional condensates. Biochimica et Biophysica Acta (BBA)-Gene Regulatory Mechanisms 2022, 1865, 194827 10.1016/j.bbagrm.2022.194827. 35618207

[ref36] Shamilov R.; Aneskievich B. J. Intrinsic disorder in nuclear receptor amino termini: from investigational challenge to therapeutic opportunity. Nuclear Receptor Research 2019, 6, 1–16. 10.32527/2019/101417.

[ref37] Kosol S.; Contreras-Martos S.; Cedeño C.; Tompa P. Structural characterization of intrinsically disordered proteins by NMR spectroscopy. Molecules 2013, 18, 10802–10828. 10.3390/molecules180910802. 24008243 PMC6269831

[ref38] Receveur-Bréchot V.; Durand D. How random are intrinsically disordered proteins? A small angle scattering perspective. Current Protein and Peptide Science 2012, 13, 55–75. 10.2174/138920312799277901. 22044150 PMC3394175

[ref39] Davidov G.; Abelya G.; Zalk R.; Izbicki B.; Shaibi S.; Spektor L.; Shagidov D.; Meyron-Holtz E. G.; Zarivach R.; Frank G. A. Folding of an intrinsically disordered iron-binding peptide in response to sedimentation revealed by cryo-EM. J. Am. Chem. Soc. 2020, 142, 19551–19557. 10.1021/jacs.0c07565. 33166133 PMC7677926

[ref40] Henriques J.; Cragnell C.; Skepo M. Molecular dynamics simulations of intrinsically disordered proteins: force field evaluation and comparison with experiment. J. Chem. Theory Comput. 2015, 11, 3420–3431. 10.1021/ct501178z. 26575776

[ref41] Salvi N.; Abyzov A.; Blackledge M. Multi-timescale dynamics in intrinsically disordered proteins from NMR relaxation and molecular simulation. journal of physical chemistry letters 2016, 7, 2483–2489. 10.1021/acs.jpclett.6b00885. 27300592

[ref42] Koder Hamid M.; Månsson L. K.; Meklesh V.; Persson P.; Skepö M. Molecular dynamics simulations of the adsorption of an intrinsically disordered protein: Force field and water model evaluation in comparison with experiments. Frontiers in Molecular Biosciences 2022, 9, 958175 10.3389/fmolb.2022.958175. 36387274 PMC9644065

[ref43] Rieloff E.; Skepö M. Molecular dynamics simulations of phosphorylated intrinsically disordered proteins: A force field comparison. International journal of molecular sciences 2021, 22, 10174 10.3390/ijms221810174. 34576338 PMC8470740

[ref44] Shapiro D. M.; Ney M.; Eghtesadi S. A.; Chilkoti A. Protein phase separation arising from intrinsic disorder: first-principles to bespoke applications. J. Phys. Chem. B 2021, 125, 6740–6759. 10.1021/acs.jpcb.1c01146. 34143622

[ref45] Bakker M. J.; Mládek A.; Semrád H.; Zapletal V.; Přecechtělová J. P. Improving IDP theoretical chemical shift accuracy and efficiency through a combined MD/ADMA/DFT and machine learning approach. Phys. Chem. Chem. Phys. 2022, 24, 27678–27692. 10.1039/D2CP01638A. 36373847

[ref46] Eliezer D. Biophysical characterization of intrinsically disordered proteins. Curr. Opin. Struct. Biol. 2009, 19, 23–30. 10.1016/j.sbi.2008.12.004. 19162471 PMC2728036

[ref47] Basu S.; Biswas P. Salt-bridge dynamics in intrinsically disordered proteins: A trade-off between electrostatic interactions and structural flexibility. Biochimica et Biophysica Acta (BBA)-Proteins and Proteomics 2018, 1866, 624–641. 10.1016/j.bbapap.2018.03.002. 29548979

[ref48] Basu S.; Mukharjee D. Salt-bridge networks within globular and disordered proteins: characterizing trends for designable interactions. J. Mol. Model. 2017, 23, 1–17. 10.1007/s00894-017-3376-y. 28626846

[ref49] Adzhubei A. A.; Sternberg M. J.; Makarov A. A. Polyproline-II helix in proteins: structure and function. Journal of molecular biology 2013, 425, 2100–2132. 10.1016/j.jmb.2013.03.018. 23507311

[ref50] Jephthah S.; Staby L.; Kragelund B.; Skepo M. Temperature dependence of intrinsically disordered proteins in simulations: What are we missing?. J. Chem. Theory Comput. 2019, 15, 2672–2683. 10.1021/acs.jctc.8b01281. 30865820

[ref51] Rath A.; Davidson A. R.; Deber C. M. The structure of “”unstructured” regions in peptides and proteins: role of the polyproline II helix in protein folding and recognition. Peptide Science: Original Research on Biomolecules 2005, 80, 179–185. 10.1002/bip.20227. 15700296

[ref52] Jephthah S.; Pesce F.; Lindorff-Larsen K.; Skepo M. Force field effects in simulations of flexible peptides with varying polyproline II propensity. J. Chem. Theory Comput. 2021, 17, 6634–6646. 10.1021/acs.jctc.1c00408. 34524800 PMC8515809

[ref53] Abraham M. J.; Murtola T.; Schulz R.; Páll S.; Smith J. C.; Hess B.; Lindahl E. GROMACS: High performance molecular simulations through multi-level parallelism from laptops to supercomputers. SoftwareX 2015, 1, 19–25. 10.1016/j.softx.2015.06.001.

[ref54] Pronk S.; Páll S.; Schulz R.; Larsson P.; Bjelkmar P.; Apostolov R.; Shirts M. R.; Smith J. C.; Kasson P. M.; Van Der Spoel D.; Hess B.; Lindahl E. GROMACS 4.5: a high-throughput and highly parallel open source molecular simulation toolkit. Bioinformatics 2013, 29, 845–854. 10.1093/bioinformatics/btt055. 23407358 PMC3605599

[ref55] Hess B.; Kutzner C.; Van Der Spoel D.; Lindahl E. GROMACS 4: algorithms for highly efficient, load-balanced, and scalable molecular simulation. J. Chem. Theory Comput. 2008, 4, 435–447. 10.1021/ct700301q. 26620784

[ref56] Berendsen H. J.; van der Spoel D.; van Drunen R. GROMACS: A message-passing parallel molecular dynamics implementation. Computer physics communications 1995, 91, 43–56. 10.1016/0010-4655(95)00042-E.

[ref57] Lindorff-Larsen K.; Piana S.; Palmo K.; Maragakis P.; Klepeis J. L.; Dror R. O.; Shaw D. E. Improved side-chain torsion potentials for the Amber ff99SB protein force field. Proteins: Struct., Funct., Bioinf. 2010, 78, 1950–1958. 10.1002/prot.22711. PMC2970904 20408171

[ref58] Hanwell M. D.; Curtis D. E.; Lonie D. C.; Vandermeersch T.; Zurek E.; Hutchison G. R. Avogadro: an advanced semantic chemical editor, visualization, and analysis platform. J. Cheminformatics 2012, 4, 1–17. 10.1186/1758-2946-4-17. PMC3542060 22889332

[ref59] Campen A.; Williams R. M.; Brown C. J.; Meng J.; Uversky V. N.; Dunker A. K. TOP-IDP-scale: a new amino acid scale measuring propensity for intrinsic disorder. Protein and peptide letters 2008, 15, 956–963. 10.2174/092986608785849164. 18991772 PMC2676888

[ref60] McGibbon R. T.; Beauchamp K. A.; Harrigan M. P.; Klein C.; Swails J. M.; Hernández C. X.; Schwantes C. R.; Wang L.-P.; Lane T. J.; Pande V. S. MDTraj: a modern open library for the analysis of molecular dynamics trajectories. Biophysical journal 2015, 109, 1528–1532. 10.1016/j.bpj.2015.08.015. 26488642 PMC4623899

[ref61] Manalastas-Cantos K.; Konarev P. V.; Hajizadeh N. R.; Kikhney A. G.; Petoukhov M. V.; Molodenskiy D. S.; Panjkovich A.; Mertens H. D.; Gruzinov A.; Borges C.; et al. ATSAS 3.0: expanded functionality and new tools for small-angle scattering data analysis. Journal of applied crystallography 2021, 54, 343–355. 10.1107/S1600576720013412. 33833657 PMC7941305

[ref62] Shen Y.; Bax A. SPARTA+: a modest improvement in empirical NMR chemical shift prediction by means of an artificial neural network. Journal of biomolecular NMR 2010, 48, 13–22. 10.1007/s10858-010-9433-9. 20628786 PMC2935510

[ref63] Schultze S.; Grubmuller H. Time-lagged independent component analysis of random walks and protein dynamics. J. Chem. Theory Comput. 2021, 17, 5766–5776. 10.1021/acs.jctc.1c00273. 34449229 PMC8444338

[ref64] Scherer M. K.; Trendelkamp-Schroer B.; Paul F.; Pérez-Hernández G.; Hoffmann M.; Plattner N.; Wehmeyer C.; Prinz J.-H.; Noé F. PyEMMA 2: A software package for estimation, validation, and analysis of Markov models. J. Chem. Theory Comput. 2015, 11, 5525–5542. 10.1021/acs.jctc.5b00743. 26574340

[ref65] Virtanen P.; Gommers R.; Oliphant T. E.; Burovski E.; Cournapeau D.; Weckesser W.; Peterson P.; van der Walt S.; Laxalde D.; Brett M.; Scipy/Scipy: Scipy 0.19. 0. Zenodo 2020.

[ref66] Danial A. Python for MATLAB Development: Extend MATLAB with 300,000+ Modules from the Python Package Index; Springer, 2022; pp 335–492.

[ref67] Jolly K. Machine learning with scikit-learn quick start guide: classification, regression, and clustering techniques in Python; Packt Publishing Ltd, 2018.

[ref68] Shahapure K. R.; Nicholas C. Cluster quality analysis using silhouette score. 2020 IEEE 7th international conference on data science and advanced analytics (DSAA); IEEE, 2020; pp 747–748.

[ref69] Hoffmann M.; Scherer M.; Hempel T.; Mardt A.; de Silva B.; Husic B. E.; Klus S.; Wu H.; Kutz N.; Brunton S. L.; et al. others Deeptime: a Python library for machine learning dynamical models from time series data. Machine Learning: Science and Technology 2022, 3, 015009 10.1088/2632-2153/ac3de0.

[ref70] Bilal S.; Iqbal H.; Anjum F.; Mir A. Prediction of 3D structure of P2RY5 gene and its mutants via comparative homology modelling. J. Comput. Biol. Bioinforma. Res. 2009, 1, 11–16.

[ref71] Nikoloudis D.; Pitts J. E.; Saldanha J. W. A complete, multi-level conformational clustering of antibody complementarity-determining regions. PeerJ. 2014, 2, e456 10.7717/peerj.456. 25071986 PMC4103072

[ref72] Bandyopadhyay A.; Basu S. Criticality in the conformational phase transition among self-similar groups in intrinsically disordered proteins: Probed by salt-bridge dynamics. Biochimica et Biophysica Acta (BBA)-Proteins and Proteomics 2020, 1868, 140474 10.1016/j.bbapap.2020.140474. 32579908

[ref73] Rieloff E.; Skepö M. The effect of multisite phosphorylation on the conformational properties of intrinsically disordered proteins. International Journal of Molecular Sciences 2021, 22, 11058 10.3390/ijms222011058. 34681718 PMC8541499

[ref74] Weinberg R. L.; Freund S. M.; Veprintsev D. B.; Bycroft M.; Fersht A. R. Regulation of DNA binding of p53 by its C-terminal domain. Journal of molecular biology 2004, 342, 801–811. 10.1016/j.jmb.2004.07.042. 15342238

[ref75] Solares M. J.; Kelly D. F. Complete Models of p53 Better Inform the Impact of Hotspot Mutations. International journal of molecular sciences 2022, 23, 15267 10.3390/ijms232315267. 36499604 PMC9740296

