# Supplementary material for: Exploring the Functional Landscape of the p53 Regulatory Domain: The Stabilizing Role of Post-Translational Modifications
Source: J Chem Theory Comput. 2024 Jul 8;20(14):5842–53. doi: 10.1021/acs.jctc.4c00570 (PMC11270737; doi:10.1021/acs.jctc.4c00570)
Supplement: Supplementary file 1 — ct4c00570_si_001.pdf [file ct4c00570_si_001.pdf]

Supporting Information for "Exploring the Functional Landscape of the p53 Regulatory Domain: The Stabilizing Role of Post-Translational Modifications"

Michael J. Bakker,[†,‡] Oskar Svensson,[‡] Henrik Vinther Sørensen,[‡,¶] and Marie Skepö*,[‡]

†*Faculty of Pharmacy in Hradec Králové, Charles University, Akademika Heyrovského 1203/8, 500 05 Hradec Králové, Czech Republic*

‡*Division of Computational Chemistry, Department of Chemistry, Lund University, P.O. Box 124, SE-221 00, Lund, Sweden*

¶*MAX IV Laboratory, Lund Institute of Advanced Neutron and X-ray Science, Scheelevägen 19, SE-223 770, Lund, Sweden*

E-mail: bakkerm@faf.cuni.cz

Table S1: EE_{DIST}, R_G, and total solvent-accessible surface area (SASA) distributions computed for their full-width half maxima (FWHM), skew, kurtosis, and respective variance at different degrees of phosphorylation.

$\mathbf{EE_{DIST}}$	$\bar{x}$ (nm)	FWHM (nm)	Skew	Kurtosis	Variance
$\mathbf{REG_{NP}}^{\ddagger}$ (conc.)	4.251	4.199	0.270	-0.443	3.180
$\mathbf{REG_{NP}}^{\ddagger}$ (lock)	3.913	3.566	0.277	-0.224	2.293
$\mathbf{REG_{NP}}^{\ddagger}$	3.614	3.319	0.352	-0.024	1.987
$\mathbf{REG_{SP}}^{\ddagger}$	3.503	3.171	0.122	-0.444	1.814
$\mathbf{REG_{FP}}^{\ddagger}$	3.123	4.333	0.793	0.050	3.386
$\mathbf{R_G}$	$\bar{x}$ (nm)	FWHM (nm)	Skew	Kurtosis	Variance
$\mathbf{REG_{NP}}^{\ddagger}$ (conc.)	4.443	6.073	0.298	-1.338	6.649
$\mathbf{REG_{NP}}^{\ddagger}$ (lock)	1.767	0.891	0.569	-0.247	0.143
$\mathbf{REG_{NP}}^{\ddagger}$	1.738	0.797	0.637	0.067	0.114
$\mathbf{REG_{SP}}^{\ddagger}$	1.719	0.692	0.559	-0.084	0.086
$\mathbf{REG_{FP}}^{\ddagger}$	1.644	0.758	0.958	0.558	0.104
**SASA**†	$\bar{x}$ (nm)	FWHM (nm)	Skew	Kurtosis	Variance
$\mathbf{REG_{NP}}^{\ddagger}$ (conc.)	67.333	11.629	1.003	0.281	24.382
$\mathbf{REG_{NP}}^{\ddagger}$ (lock)	55.705	5.185	-0.104	-0.529	4.847
$\mathbf{REG_{NP}}^{\ddagger}$	59.097	5.072	0.077	-0.445	4.639
$\mathbf{REG_{SP}}^{\ddagger}$	58.099	6.494	0.064	-0.793	7.604
$\mathbf{REG_{FP}}^{\ddagger}$	57.125	5.553	0.267	-0.153	5.559

† - Total solvent-accessible surface areas (SASAs) only computed from non-phosphorylated residues.
‡ - NP/SP/FP = non-phosphorylated, single-phosphorylated, and full-phosphorylated, respectively.

Table S2: P-values computed between each observed radius of gyration (R_G), end-to-end distances (EE_{dist}) and solvent-accessible surface area (SASA) in the non-phosphorylated (REG_{NP}), single-phosphorylated (REG_{SP}), and fully-phosphorylated (REG_{FP}) trajectories.

Trajectories	$\mathbf{R_G}$ (nm)	$\mathbf{EE_{dist}}$ (nm)	Total SASA (nm^2)
$\mathbf{REG_{NP}}$ / $\mathbf{REG_{SP}}$	1.645e-3	3.19e-05	1.08e-23
$\mathbf{REG_{NP}}$ / $\mathbf{REG_{FP}}$	2.93e-45	0.00	3.04e-131
$\mathbf{REG_{SP}}$ / $\mathbf{REG_{FP}}$	7.01e-33	1.75e-32	3.45e-268

Table S3: Total SASA distributions computed for their FWHM, skew, kurtosis, and respective variance at different phosphorylation degrees, including the phosphorylated residue.

Total SASA	$\bar{x}$ (nm)	FWHM (nm)	Skew	Kurtosis	Variance
$\mathbf{REG_{NP}}$	54.68	9.48	-0.36	-0.11	16.22
$\mathbf{REG_{SP}}$	53.91	8.36	-0.03	-0.01	12.61
$\mathbf{REG_{FP}}$	56.70	9.77	-0.41	-0.05	17.22

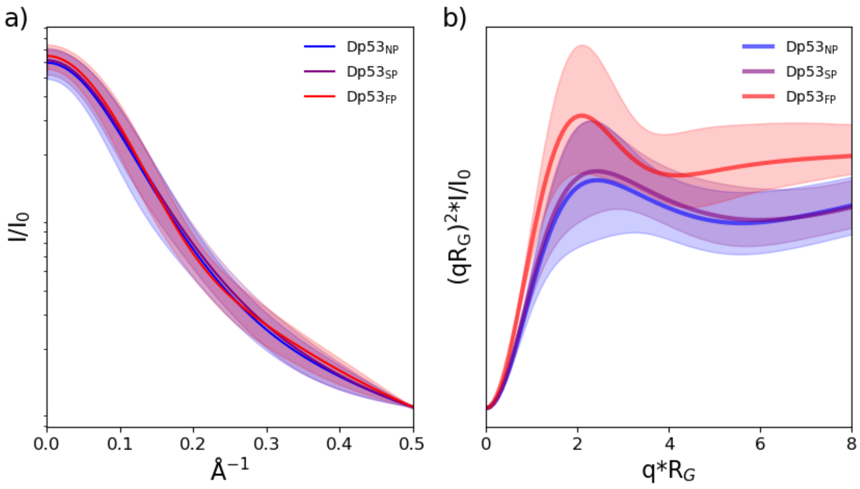

Figure S1: Form factor (a) and dimensionless Kratky plot (b) of the different levels of phosphorylation on the REG of p53.

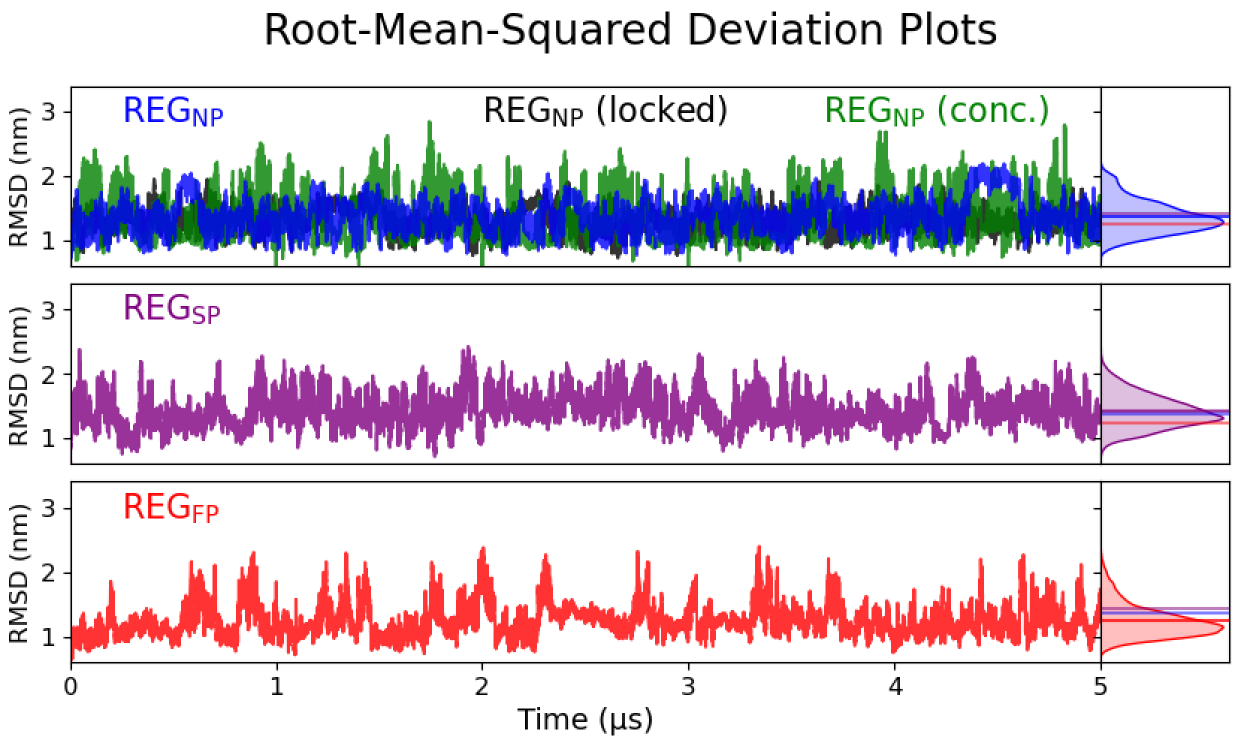

Figure S2: The root-mean-squared deviation (RMSD) for each of the trajectories projected onto a kernel-density estimation (KDE) plot to show the variability in structures upon different levels of phosphorylation.

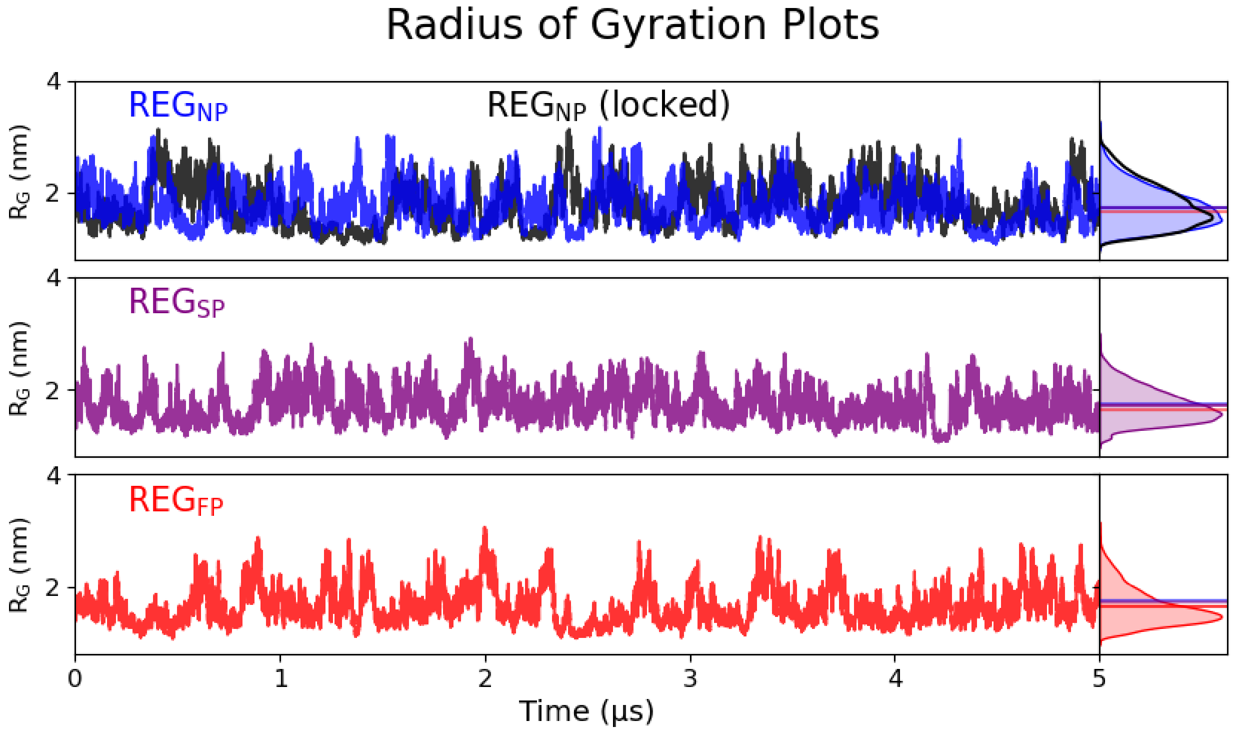

Figure S3: The radius of gyration (R_G) for each of the trajectories projected onto a kernel-density estimation (KDE) plot to show the variability in structures upon different levels of phosphorylation.

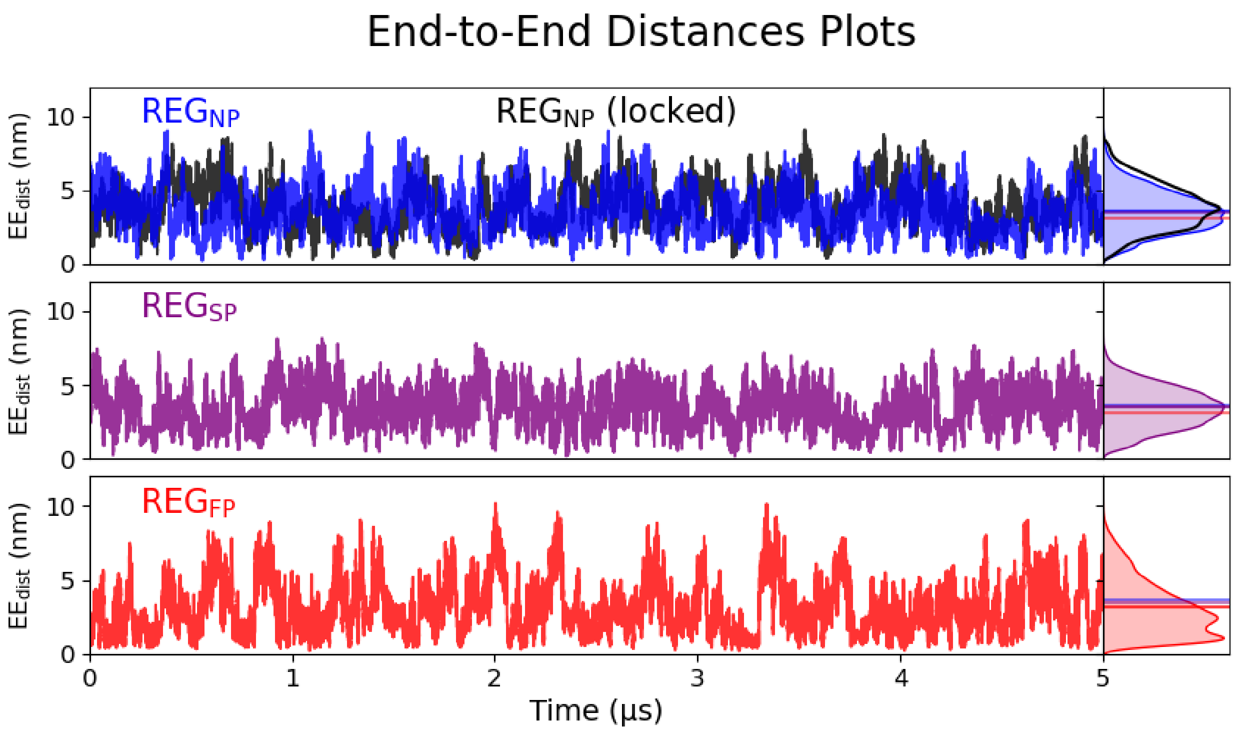

Figure S4: The end-to-end distance (EE_{DIST}) for each of the trajectories projected onto a kernel-density estimation (KDE) plot to show the variability in structures upon different levels of phosphorylation.

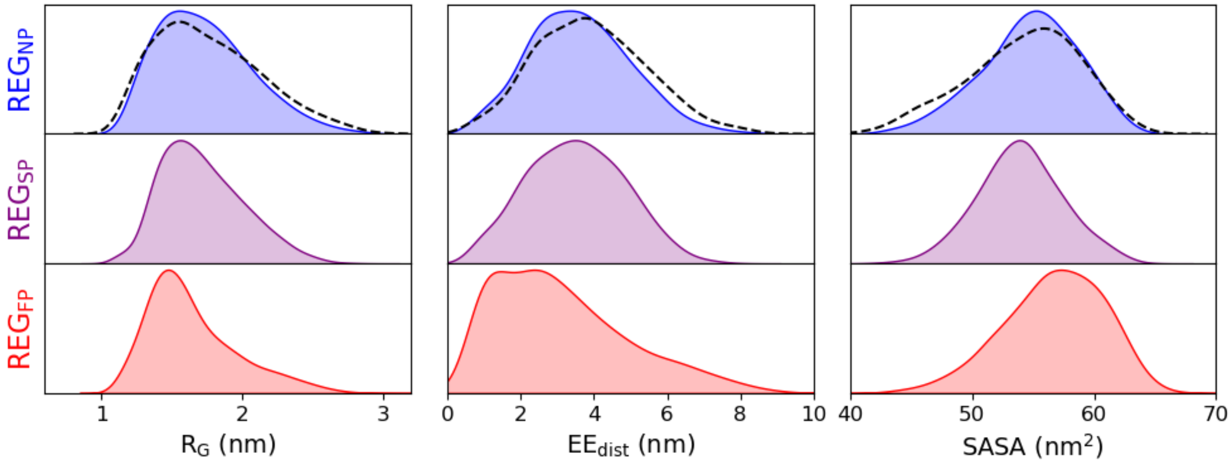

Figure S5: The R_G (nm), EE_{dist}, and total SASA for each trajectory in the REG of p53 shown as kernel-density estimations (KDE) plots as well as the locked counterpart in the non-phosphorylated trajectory (dashed), with vertical lines indicating the mean.

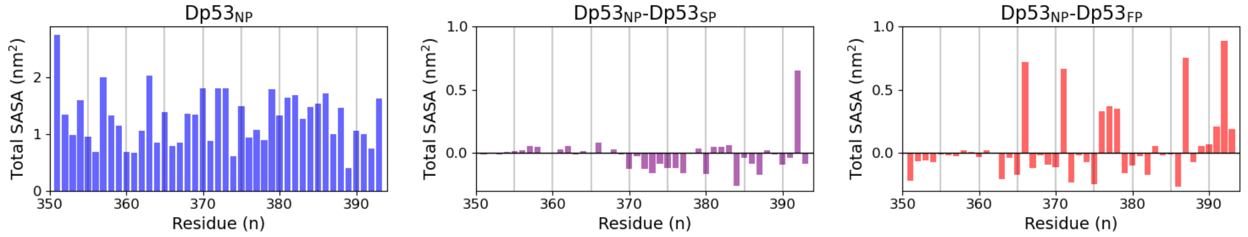

Figure S6: Total SASA per residue in the non-phosphorylated (a), and changes in the single phosphorylated (b), and fully phosphorylated (c) trajectories.

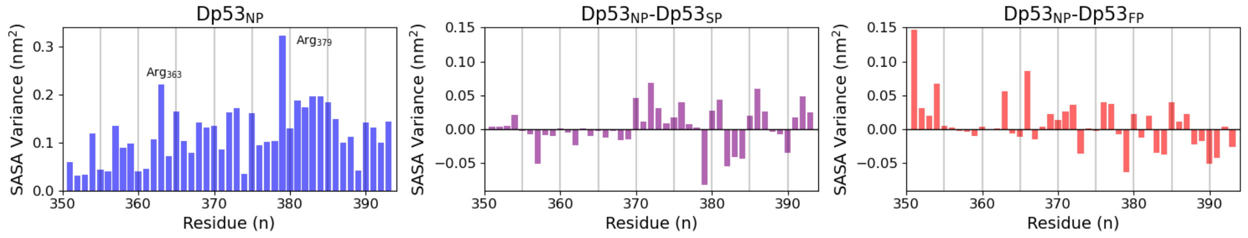

Figure S7: Total SASA variance per residue in the non-phosphorylated (a), single phosphorylated (b), and fully phosphorylated (c) trajectories, with specific residues labelled for focus. **Replace Dp53 with REG.**

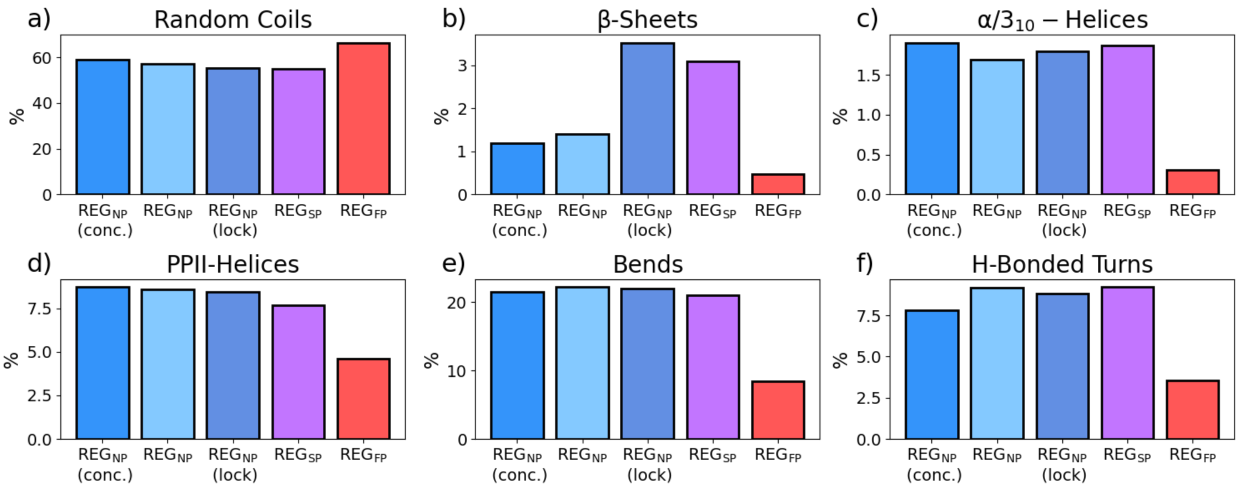

Figure S8: Instances of secondary structure in the regulatory domain (REG) in the non-phosphorylated, REG_{NP}, non-phosphorylated restrained, REG_{NP}^{lock}, non-phosphorylated high concentration, $REG_{NP}^{conc.}$, single-phosphorylated, REG_{SP}, and fully-phosphorylated, REG_{FP}, trajectories.

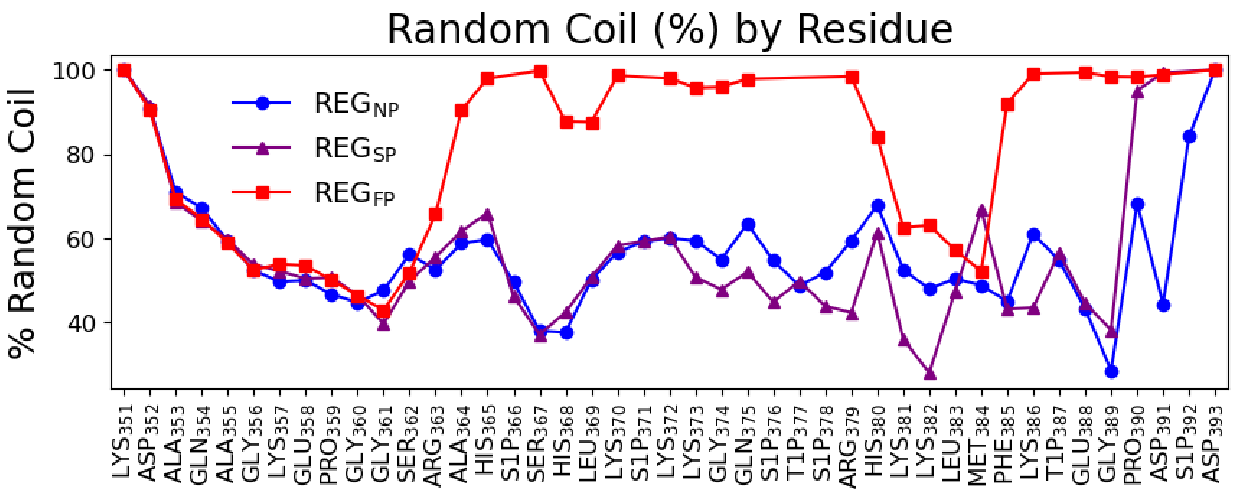

Figure S9: Include caption.

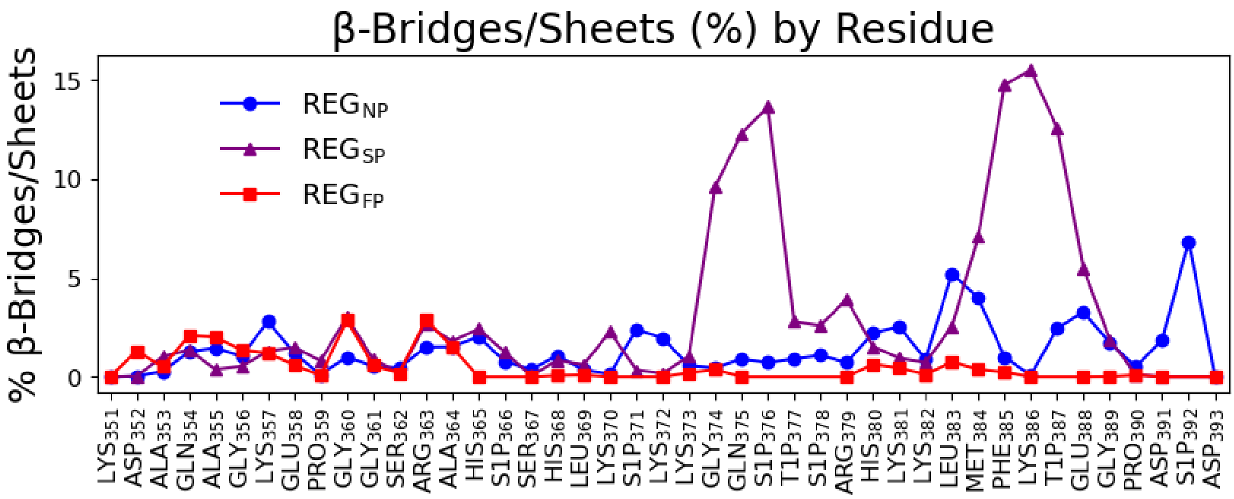

Figure S10: Include caption.

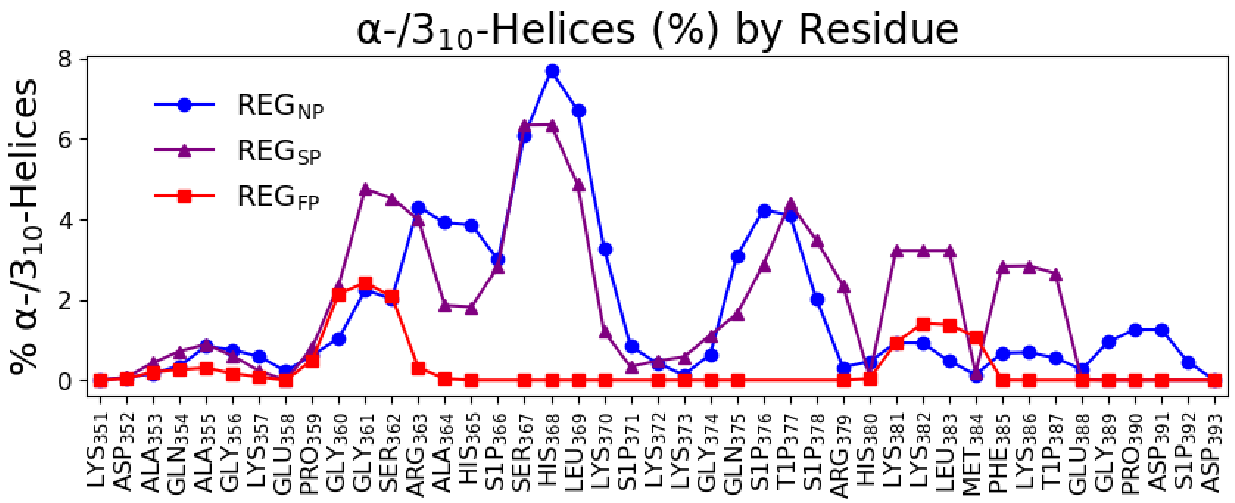

Figure S11: Include caption.

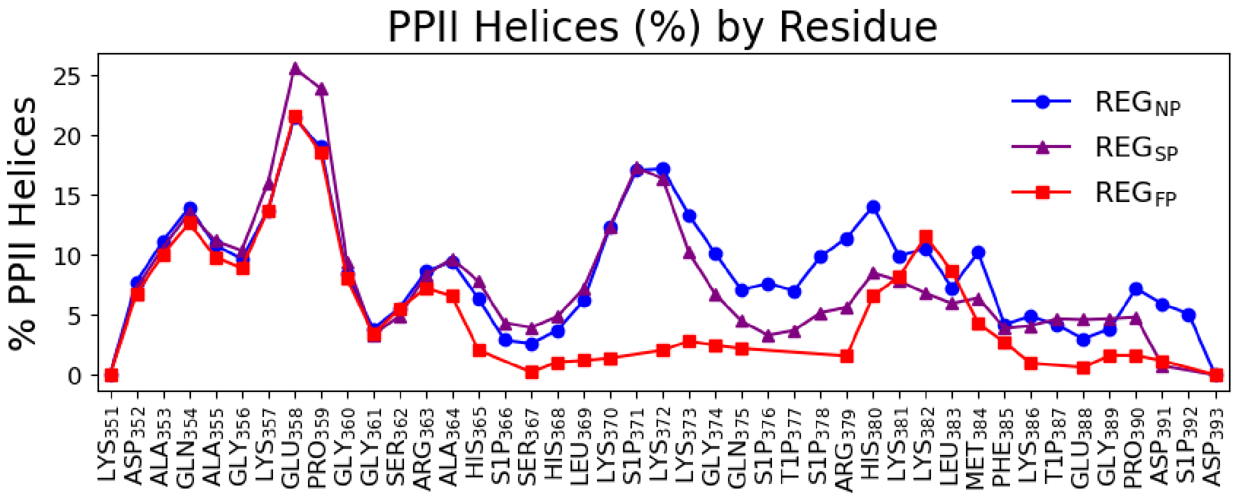

Figure S12: Include caption.

Table S4: Comparison of cluster globular properties of the protein REG_{NP}. The table presents the percentage of each cluster in each trajectory of $REG_{351-393}$ (NP, SP, and FP) in the dataset, along with the mean and standard deviation of the radius of gyration (R_G), end-to-end distances (EE_{DIST}), and total solvent accessible surface area (SASA). The results are sorted in descending order by the percentage of each trajectory, and the overall globular trends are shown at the bottom.

**Trajectory**	**(n)**	**%**	**R_G (nm)**	**SASA (nm^2)**	**EE_{DIST} (nm)**
	1	19.4 %	1.72 ± 0.34	54.4 ± 3.79	3.79 ± 1.43
	2	23.6 %	1.61 ± 0.31	52.2 ± 4.03	3.27 ± 1.30
REG_{NP}	3	55.3 %	1.79 ± 0.33	55.7 ± 3.63	3.68 ± 1.42
	4	1.6 %	1.82 ± 0.32	54.9 ± 3.07	3.97 ± 1.23
	ALL		1.73 ± 0.33	54.6 ± 4.02	3.61 ± 1.41
	1	23.6 %	1.68 ± 0.26	53.4 ± 3.55	3.77 ± 1.23
	2	12.5 %	1.61 ± 0.34	51.5 ± 4.37	3.20 ± 1.57
REG_{NP}^{lock}	3	56.4 %	1.88 ± 0.38	55.9 ± 3.96	4.22 ± 1.56
	4	7.35 %	1.35 ± 0.17	47.4 ± 4.08	3.19 ± 1.04
	ALL		1.76 ± 0.37	54.1 ± 4.64	3.91 ± 1.51
	1	13.0 %	1.69 ± 0.34	55.0 ± 4.82	3.45 ± 1.52
	2	19.2 %	1.74 ± 0.31	55.3 ± 3.32	3.58 ± 1.32
	3	19.8 %	1.71 ± 0.26	53.5 ± 3.15	3.79 ± 1.23
REG_{SP}	4	11.8 %	1.63 ± 0.31	53.8 ± 3.45	3.33 ± 1.44
	5	12.5 %	1.70 ± 0.27	53.5 ± 2.71	3.43 ± 1.19
	6	23.5 %	1.76 ± 0.25	52.6 ± 3.03	3.32 ± 1.30
	ALL		1.71 ± 0.29	53.9 ± 3.55	3.50 ± 1.34
	1	13.4 %	1.60 ± 0.30	56.4 ± 3.96	2.88 ± 1.70
REG_{FP}	2	13.4 %	1.60 ± 0.23	56.6 ± 3.22	2.70 ± 1.36
	3	73.1 %	1.66 ± 0.33	56.7 ± 4.33	3.23 ± 1.91
	ALL		1.64 ± 0.32	56.7 ± 4.15	3.11 ± 1.83

Table S5: Comparison of cluster globular properties of $REG_{351-393}$. The table presents the percentage of each cluster in each trajectory of $REG_{351-393}$ (NP, SP, and FP) in the dataset, along with the mean and standard deviation of the radius of gyration (R_G), end-to-end distances (EE_{DIST}), and total solvent accessible surface area (SASA). The results are sorted in descending order by the percentage of each trajectory, and the overall globular trends are shown at the bottom.

**Trajectory**	**(n)**	**%**	**Coil**	**β-Sheets**	**PPII-Helices**	**α-Helices**
REG_{NP}	1	19.4 %	23.81	0.485	3.237	1.197
	2	23.6 %	22.82	1.169	3.815	0.725
	3	55.3 %	25.45	0.402	3.783	0.516
	4	1.6 %	22.71	0.325	5.062	0.15
	ALL		24.46	0.598	3.705	0.692
REG_{NP}^{lock}	1	23.6 %	23.40	1.139	3.229	0.886
	2	12.5 %	21.81	2.798	3.009	1.168
	3	56.4 %	25.02	0.712	4.111	0.673
	4	7.35 %	18.83	6.929	2.725	0.480
	ALL		23.78	1.532	3.663	0.771
REG_{SP}	1	13.0 %	24.31	0.676	3.724	0.743
	2	19.2 %	24.11	0.864	3.628	0.720
	3	19.8 %	23.78	0.131	2.996	0.928
	4	11.8 %	25.02	0.342	2.825	0.384
	5	12.5 %	22.97	0.472	2.866	2.146
	6	23.5 %	22.43	3.990	3.325	0.297
	ALL		23.64	1.320	3.253	0.804
REG_{FP}	1	13.4 %	27.35	0.314	1.652	0.171
	2	13.4 %	27.89	0.252	1.787	0.078
	3	73.1 %	28.86	0.186	2.108	0.133
	ALL		28.53	0.212	2.004	0.131

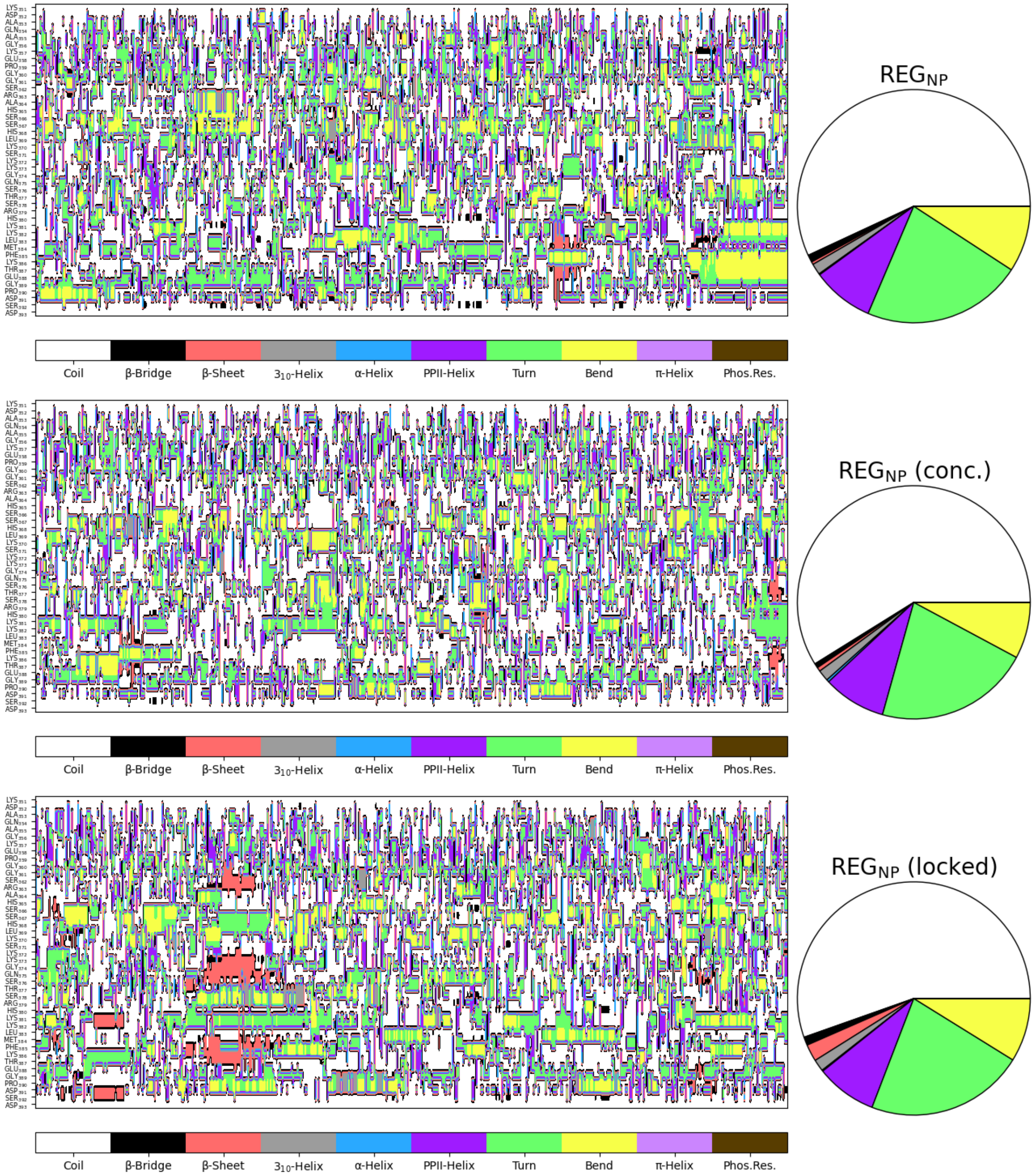

Figure S13: DSSP plots for each of the trajectories; (top) non-phosphorylated, $\mathrm{REG}_{\mathrm{NP}}$, (middle) non-phosphorylated locked, $\mathrm{REG}_{\mathrm{NP}}^{\mathrm{lock}}$, and (bottom) non-phosphorylated concentrated, $\mathrm{REG}_{\mathrm{NP}}^{\mathrm{conc.}}$.

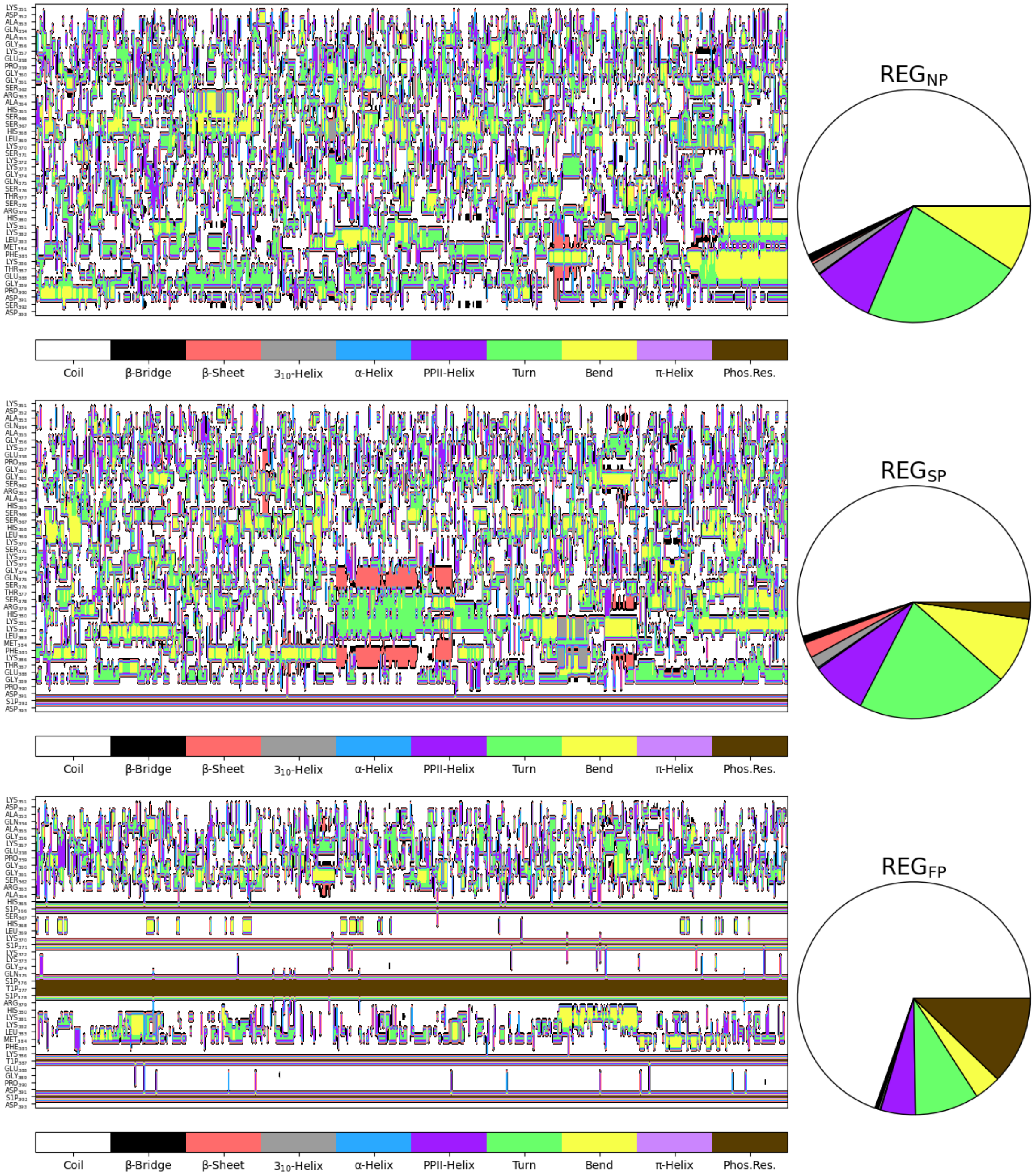

Figure S14: DSSP plots for each of the trajectories; (top) non-phosphorylated, REG$_{\mathrm{NP}}$, (middle) single-phosphorylated, REG$_{\mathrm{SP}}$, and (bottom) fully phosphorylated, REG$_{\mathrm{FP}}$.

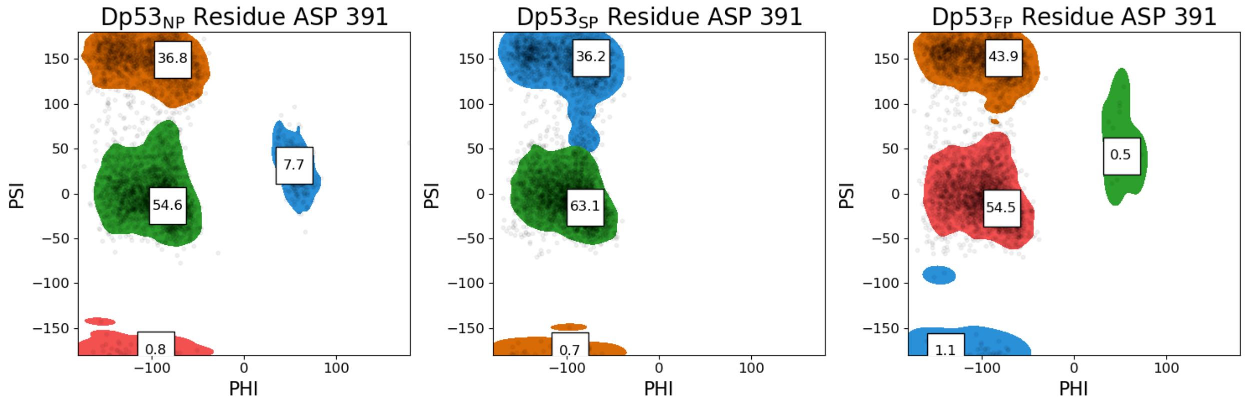

Figure S15: Ramachandran plots in residue ASP_{391} for REG_{NP} (left), REG_{SP} (center), and REG_{FP} (right) with specific clustered regions and the percentage of the trajectory that exists in each region.

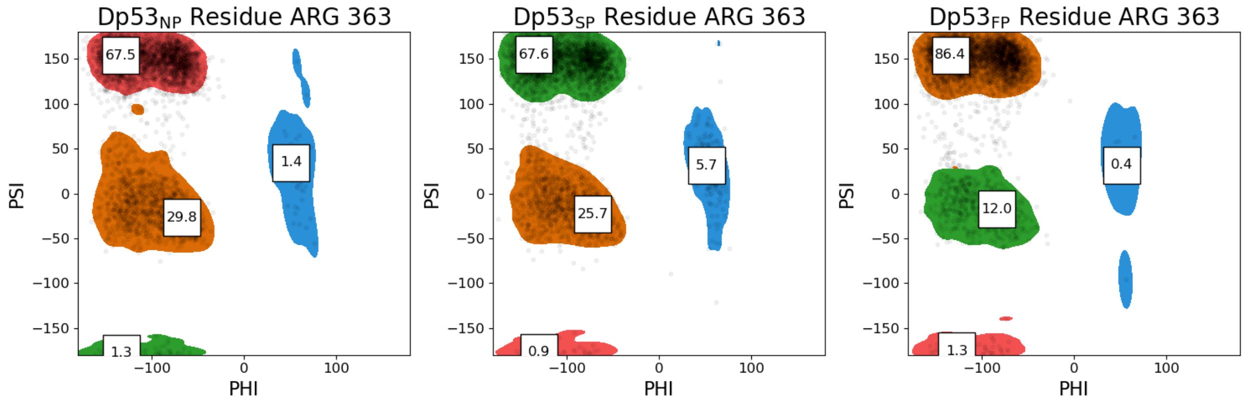

Figure S16: Ramachandran plots in residue ARG_{363} for REG_{NP} (left), REG_{SP} (center), and REG_{FP} (right) with specific clustered regions and the percentage of the trajectory that exists in each region.

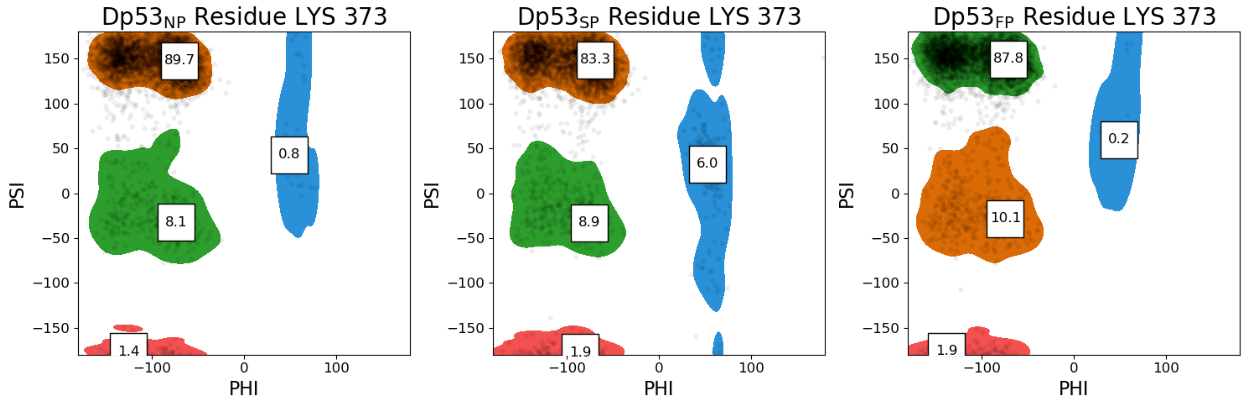

Figure S17: Ramachandran plots in residue LYS_{373} for REG_{NP} (left), REG_{SP} (center), and REG_{FP} (right) with specific clustered regions and the percentage of the trajectory that exists in each region.

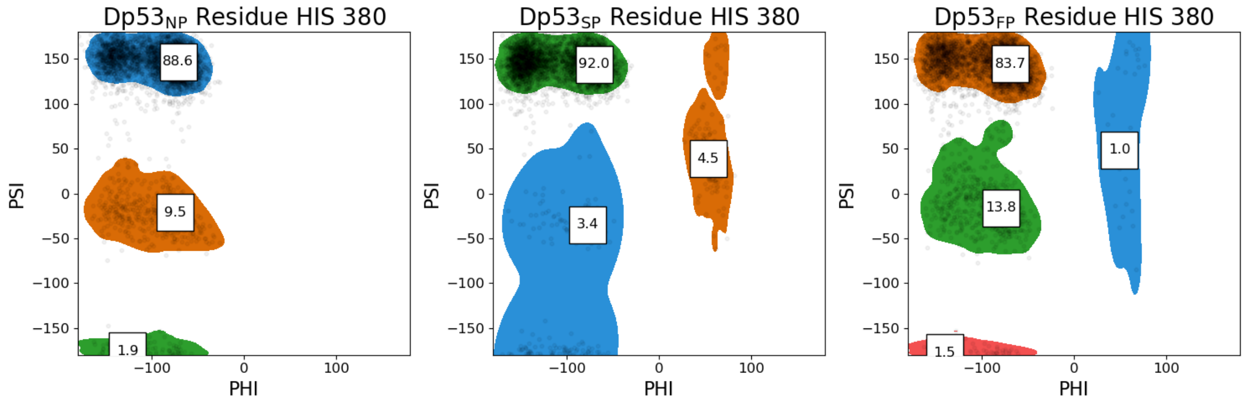

Figure S18: Ramachandran plots in residue HIS_{380} for REG_{NP} (left), REG_{SP} (center), and REG_{FP} (right) with specific clustered regions and the percentage of the trajectory that exists in each region.

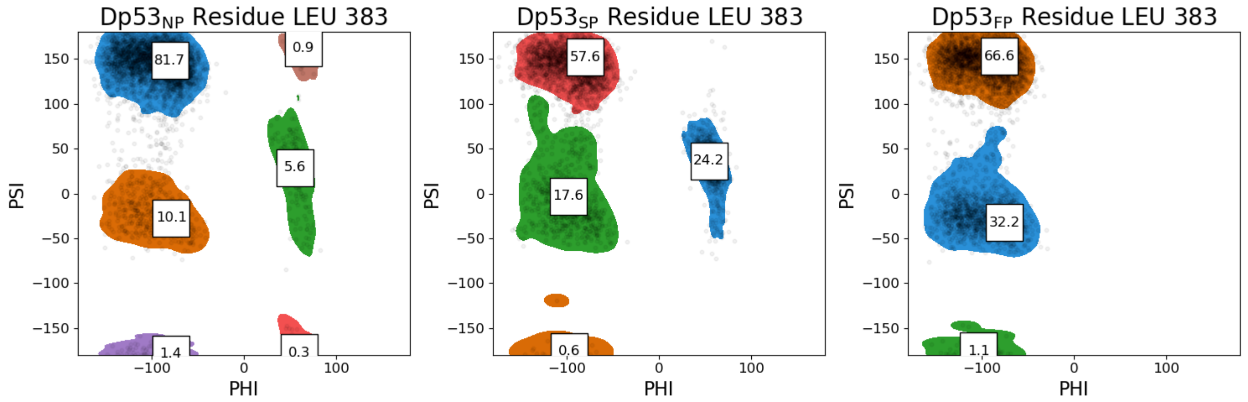

Figure S19: Ramachandran plots in residue LEU_{383} for REG_{NP} (left), REG_{SP} (center), and REG_{FP} (right) with specific clustered regions and the percentage of the trajectory that exists in each region.

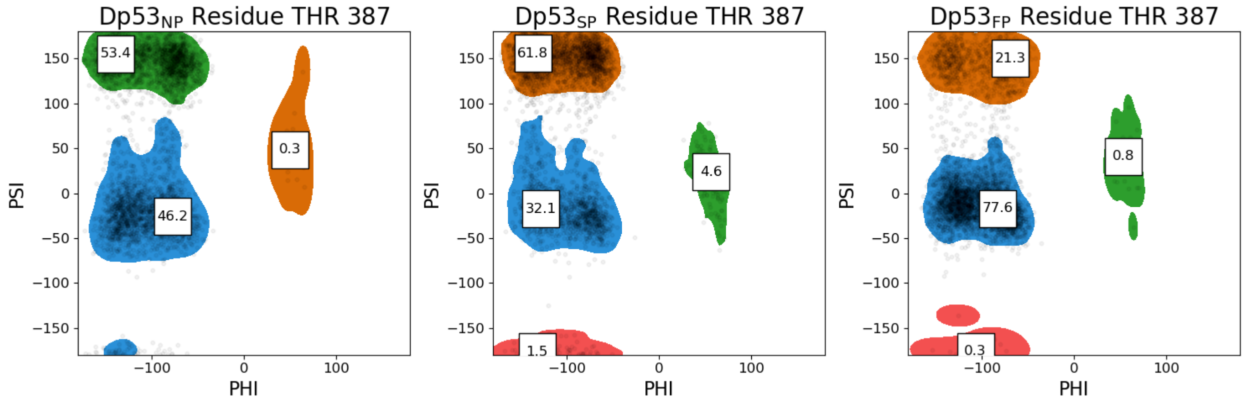

Figure S20: Ramachandran plots in residue THR_{387} for REG_{NP} (left), REG_{SP} (center), and REG_{FP} (right) with specific clustered regions and the percentage of the trajectory that exists in each region.

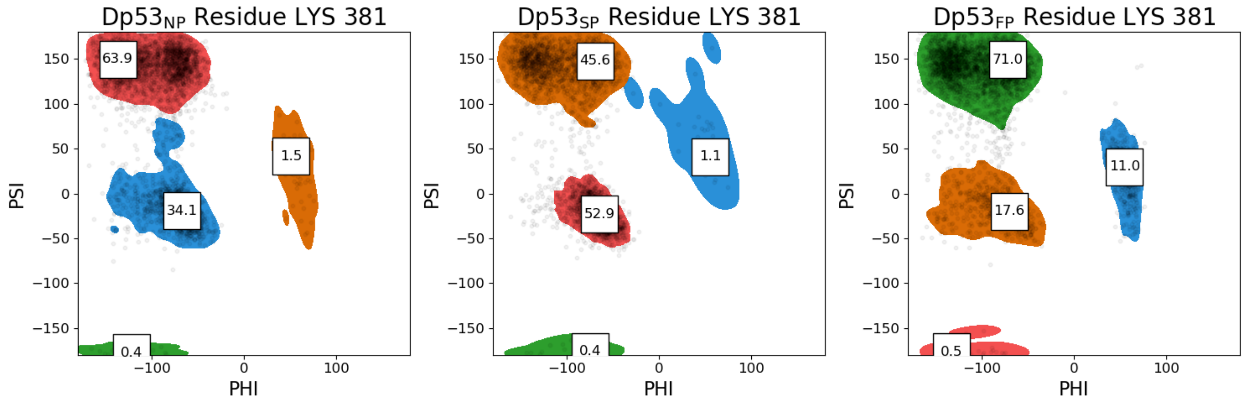

Figure S21: Ramachandran plots in residue LYS_{381} for REG_{NP} (left), REG_{SP} (center), and REG_{FP} (right) with specific clustered regions and the percentage of the trajectory that exists in each region.

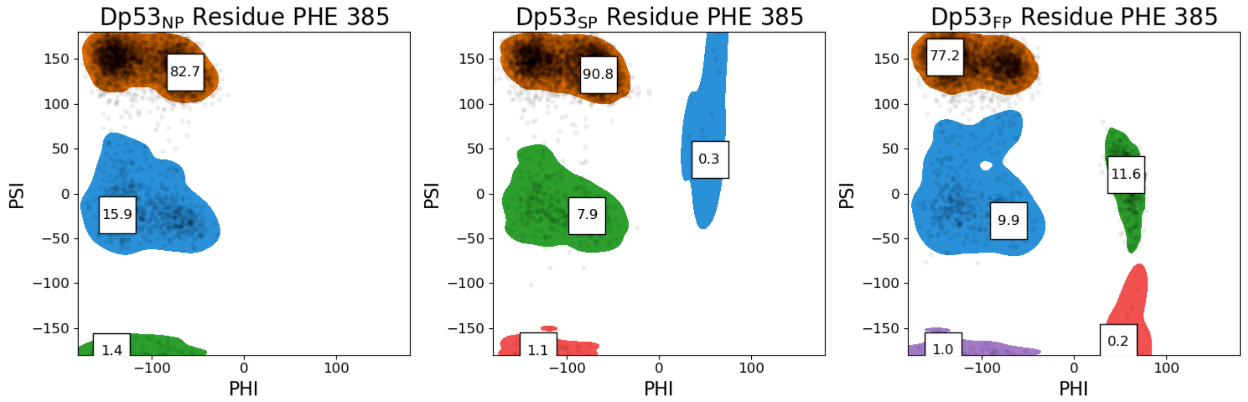

Figure S22: Ramachandran plots in residue PHE_{385} for REG_{NP} (left), REG_{SP} (center), and REG_{FP} (right) with specific clustered regions and the percentage of the trajectory that exists in each region.

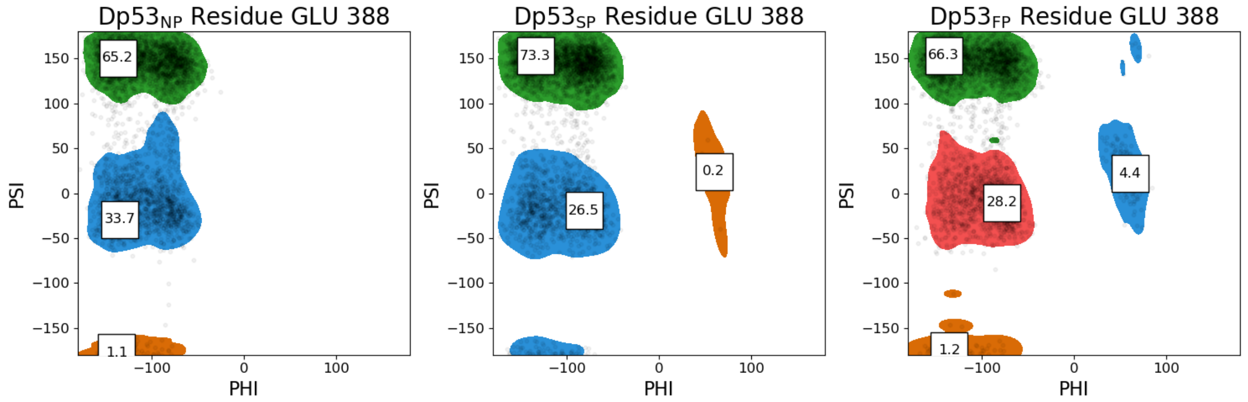

Figure S23: Ramachandran plots in residue GLU_{388} for REG_{NP} (left), REG_{SP} (center), and REG_{FP} (right) with specific clustered regions and the percentage of the trajectory that exists in each region.

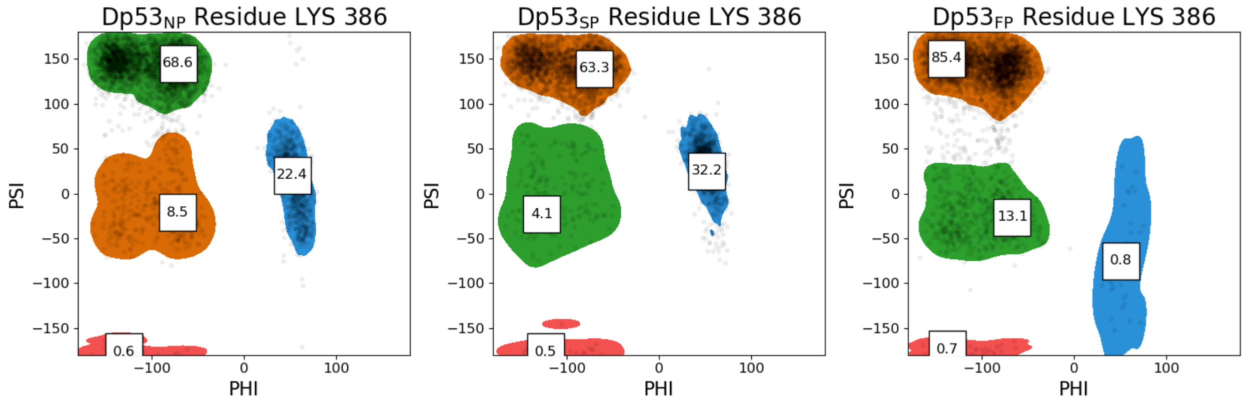

Figure S24: Ramachandran plots in residue LYS_{386} for REG_{NP} (left), REG_{SP} (center), and REG_{FP} (right) with specific clustered regions and the percentage of the trajectory that exists in each region.

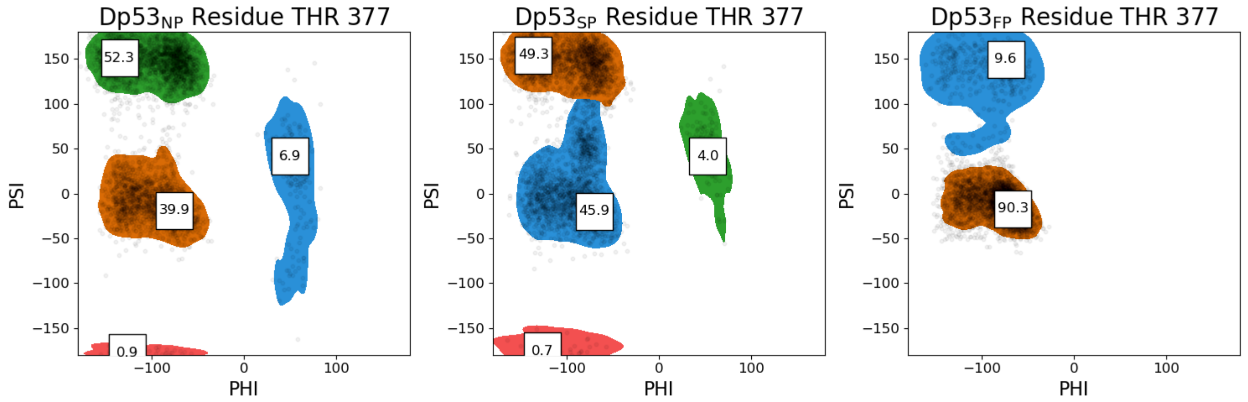

Figure S25: Ramachandran plots in residue THR_{377} for REG_{NP} (left), REG_{SP} (center), and REG_{FP} (right) with specific clustered regions and the percentage of the trajectory that exists in each region.

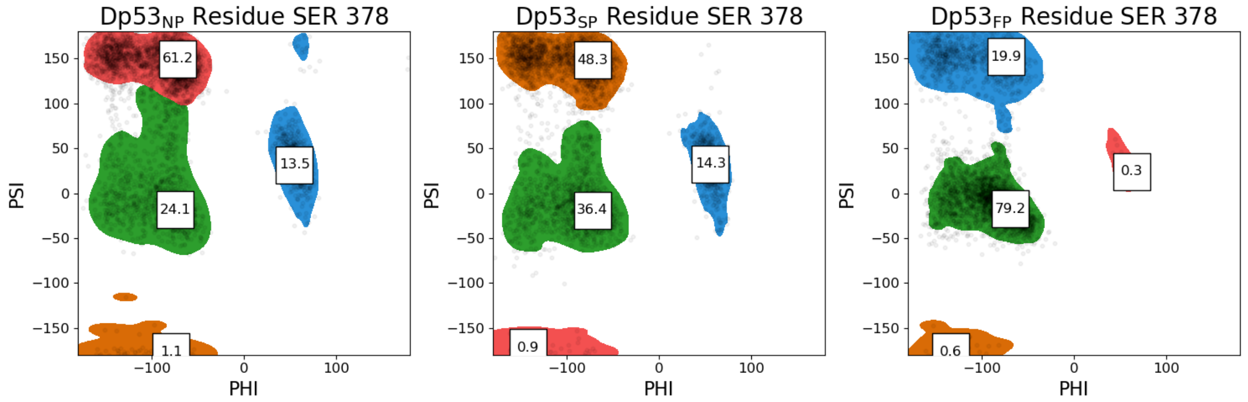

Figure S26: Ramachandran plots in residue SER_{378} for REG_{NP} (left), REG_{SP} (center), and REG_{FP} (right) with specific clustered regions and the percentage of the trajectory that exists in each region.

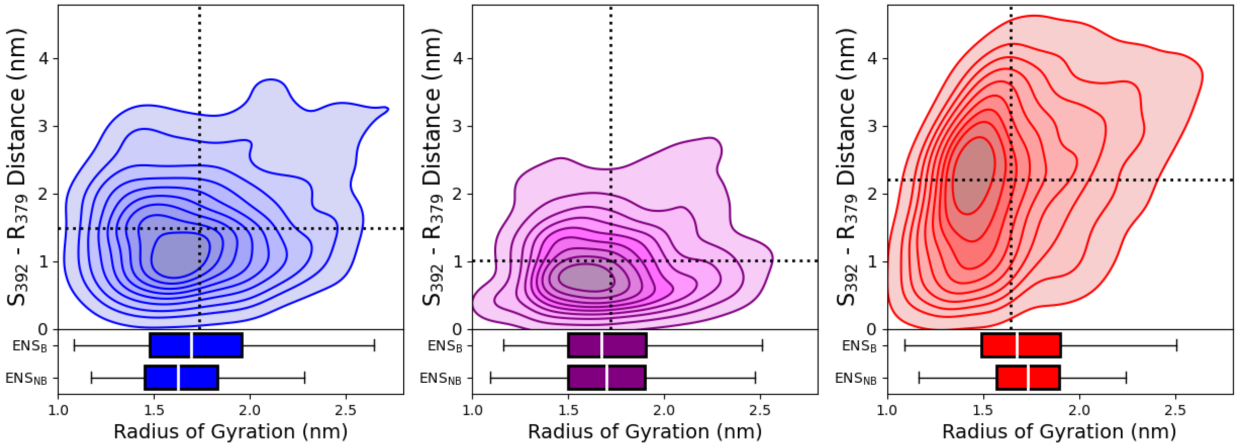

Figure S27: Bivariate density plot representing the distribution of the radius of gyration and as a function of the distance between residues S_{392} and R_{379}, with representative box plots showing the influence on the radius gyration in ensembles in which they are bonded (top), ENS_B and non-bonded (bottom), ENS_{NB} for the trajectories non-phosphorylated (blue), single phosphorylated (purple) and fully phosphorylated (red).

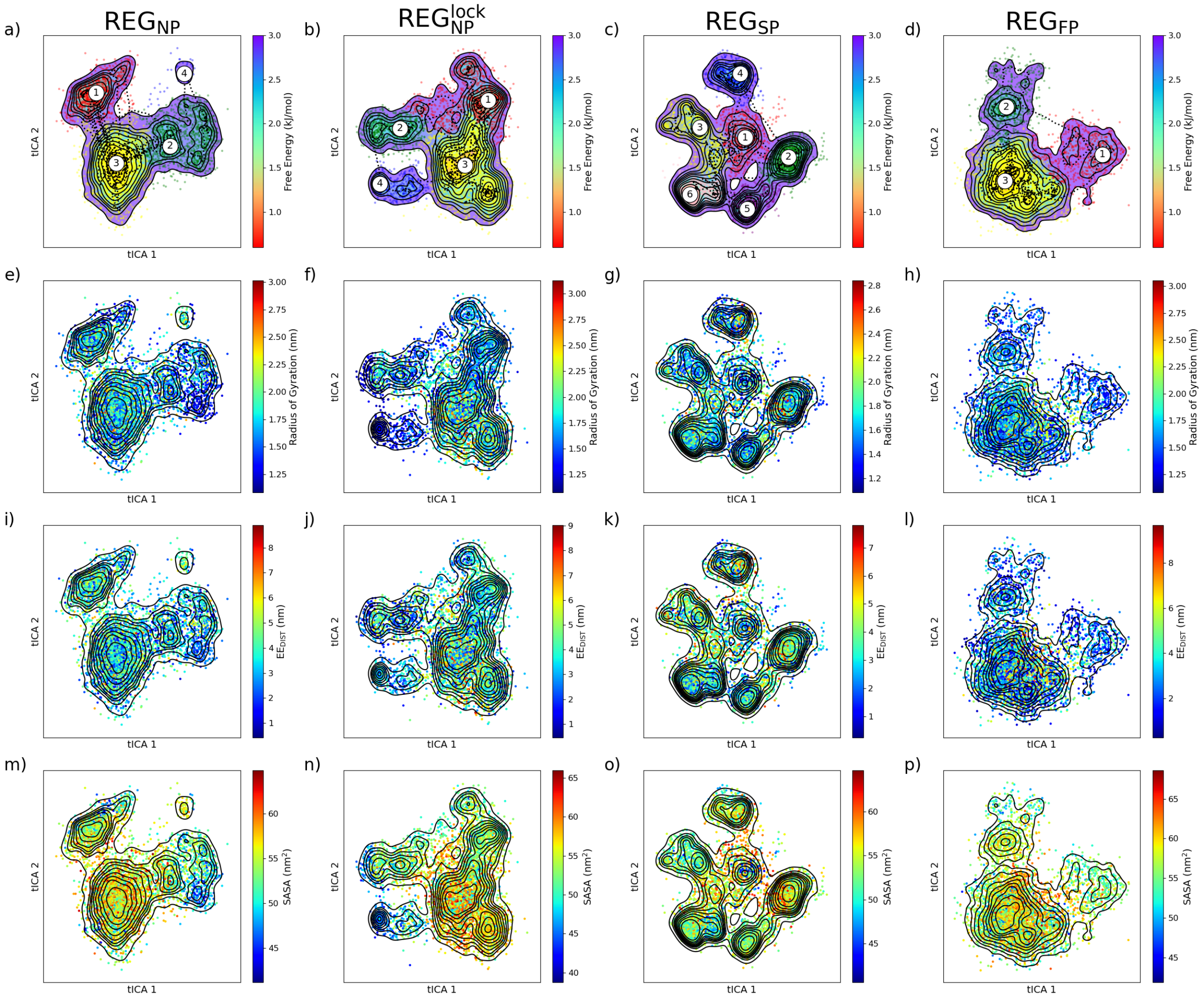

Figure S28: Free energy plots were generated using time-lagged independent component analysis, tICA, dimensionality reduction on ϕ and ψ angles (a-d) in $\mathrm{REG_{NP}}$ (a), $\mathrm{REG_{NP}^{locked}}$ (b), $\mathrm{REG_{SP}}$ (c), and $\mathrm{REG_{FP}}$ (d) with centers identified by agglomerative hierarchical clustering and the Silhouette score (blue) and Davies Bouldin index (red) displayed as a function of cluster size (e-h), as well as the cluster distributions for the maximum Silhouette score clustering (i-l) and the minimum Davies Bouldin index (m-p).

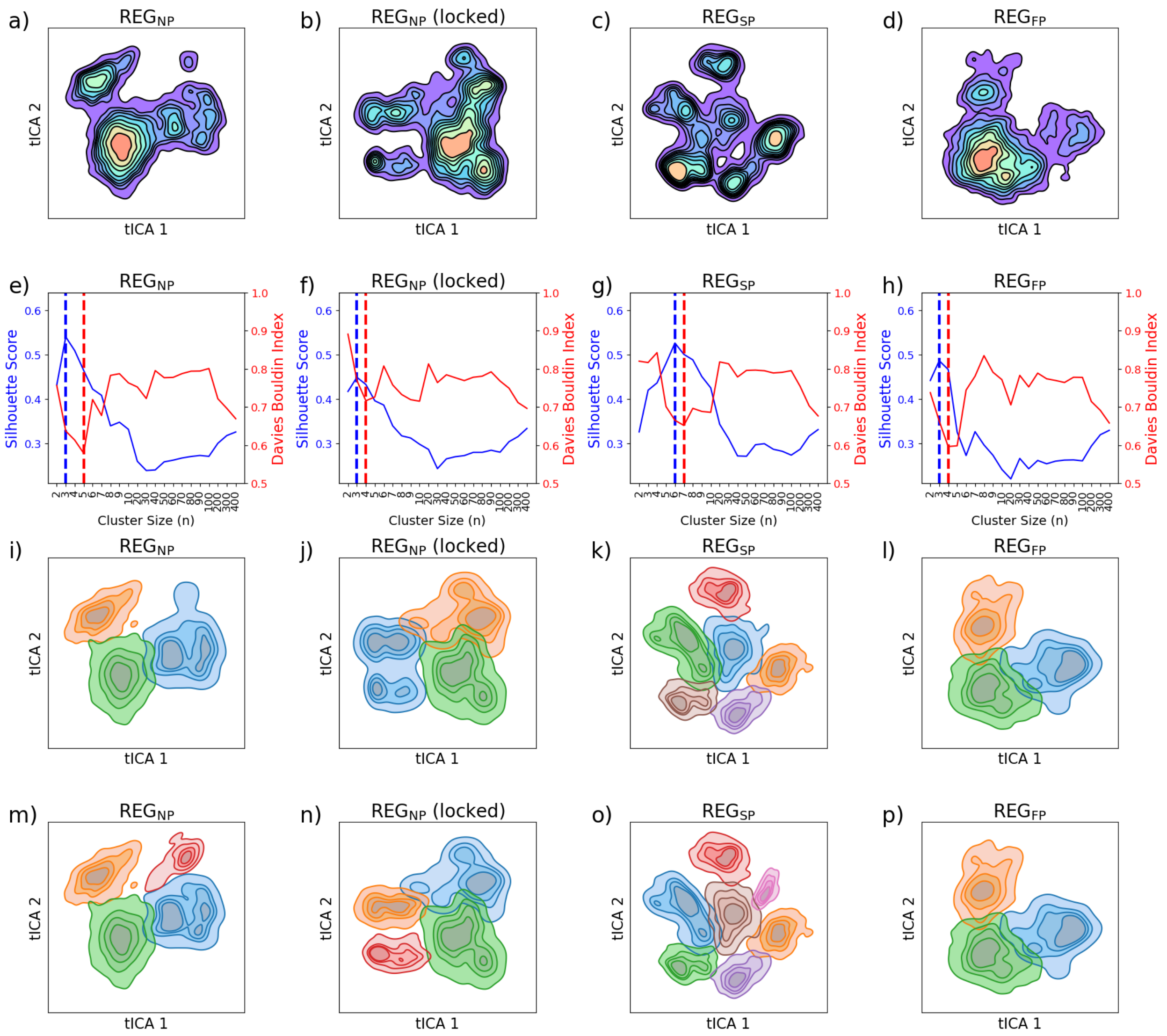

Figure S29: Free energy plots were generated using tICA dimensionality reduction on ϕ and ψ angles (a-d) in $\mathrm{REG_{NP}}$ (a), $\mathrm{REG_{NP}^{locked}}$ (b), $\mathrm{REG_{SP}}$ (c), and $\mathrm{REG_{FP}}$ (d) with centers identified by agglomerative hierarchical clustering and the Silhouette score (blue) and Davies Bouldin index (red) displayed as a function of cluster size (e-h), as well as the cluster distributions for the maximum Silhouette score clustering (i-l) and the minimum Davies Bouldin index (m-p).

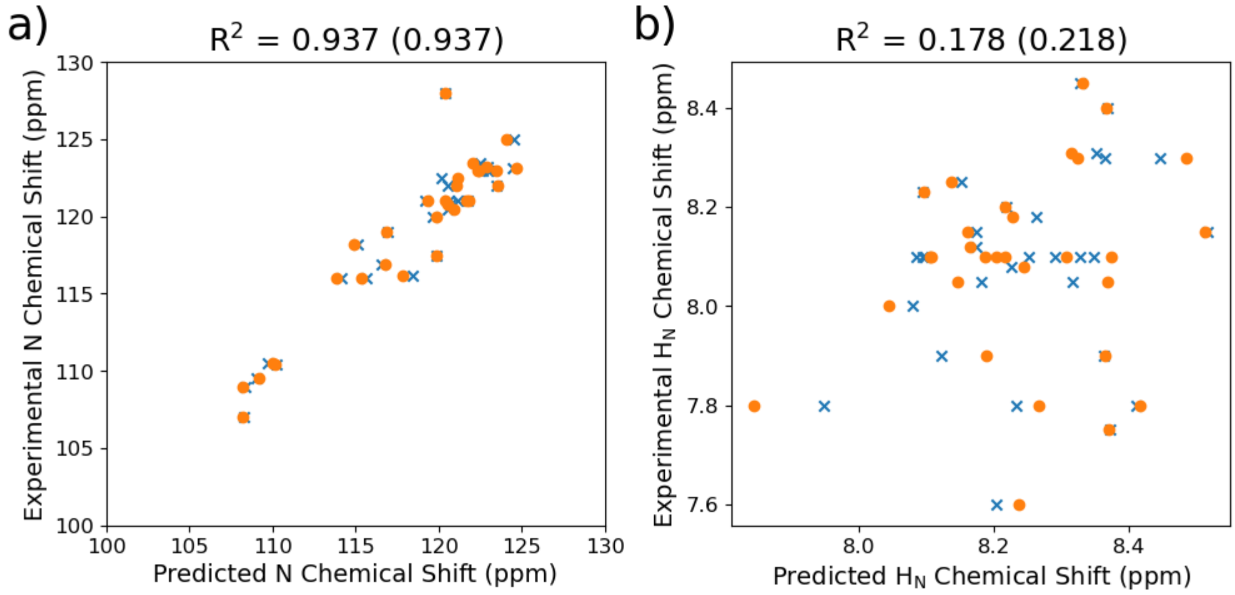

Figure S30: Comparison of the chemical shifts computed by Sparta+ from the non-phosphorylated trajectory to experimental values, as well as the r^2 values for each, with the restrained trajectory in ().

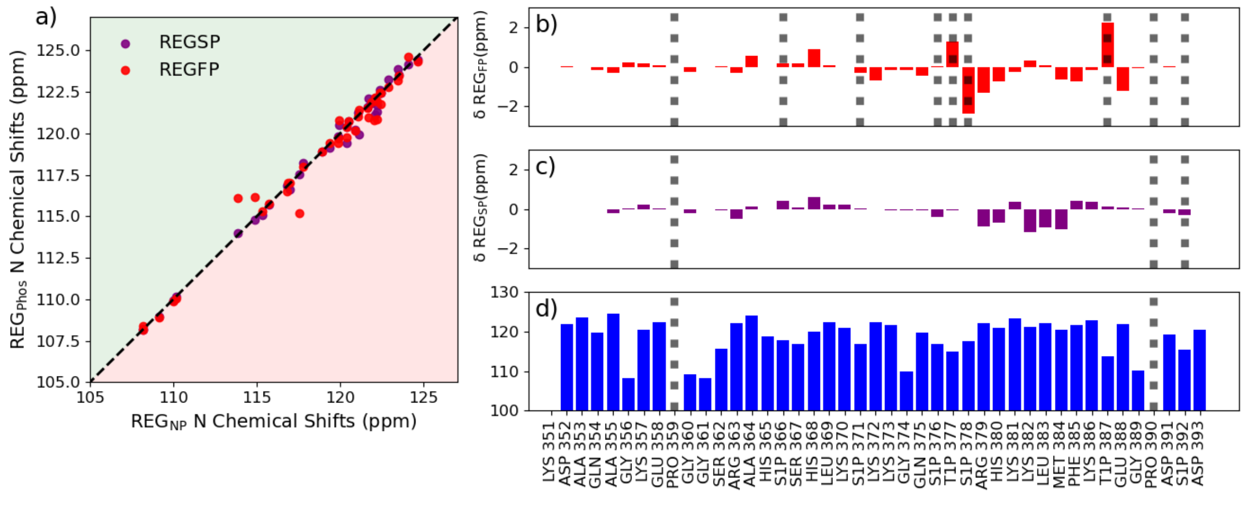

Figure S31: (a) Correlation plot between the phosphorylated and non-phosphorylated predicted chemical shifts as well as (d) a barchart of the chemical shifts from the non-phosphorylated trajectory and the change in chemical shifts in the (c) single-phosphorylated trajectory and the (d) fully-phosphorylated trajectory.
